# Review of 128 quality of care mechanisms: A framework and mapping for health system stewards

**DOI:** 10.1016/j.healthpol.2019.11.006

**Published:** 2020-01

**Authors:** Juan E. Tello, Erica Barbazza, Kerry Waddell

**Affiliations:** a Integrated Prevention and Control of NCDs Programme, Division of NCDs and Promoting Health through the Life-Course, WHO Regional Office for Europe, Copenhagen, Denmark; b Academic UMC, Department of Public Health, University of Amsterdam, Amsterdam Public Health Research Institute, Amsterdam, the Netherlands; c McMaster Health Forum, McMaster University, Hamilton, Canada; d WHO European Centre for Primary Health Care, Almaty, Kazakhstan

**Keywords:** Quality of health care, Quality improvement, Health policy, Clinical governance

## Abstract

Health system stewards have the critical task to identify quality of care deficiencies and resolve underlying system limitations. Despite a growing evidence-base on the effectiveness of certain mechanisms for improving quality of care, frameworks to facilitate the oversight function of stewards and the use of mechanisms to improve outcomes remain underdeveloped. This review set out to catalogue a wide range of quality of care mechanisms and evidence on their effectiveness, and to map these in a framework along two dimensions: (i) governance subfunctions; and (ii) targets of quality of care mechanisms. To identify quality of care mechanisms, a series of searches were run in Health Systems Evidence and PubMed. Additional grey literature was reviewed. A total of 128 quality of care mechanisms were identified. For each mechanism, searches were carried out for systematic reviews on their effectiveness. These findings were mapped in the framework defined. The mapping illustrates the range and evidence for mechanisms varies and is more developed for some target areas such as the health workforce. Across the governance sub-functions, more mechanisms and with evidence of effectiveness are found for setting priorities and standards and organizing and monitoring for action. This framework can support system stewards to map the quality of care mechanisms used in their systems and to uncover opportunities for optimization backed by systems thinking.

## Highlights

• Quality of care mechanisms are available across governance sub-functions and target areas.
• Most of the 128 mechanisms relate to setting priorities/standards and organizing/monitoring action.
• The taxonomy developed can serve as a tool for prioritization by system stewards.
• 13 mechanisms have sufficient evidence on their effectiveness, 33 have some evidence.
• The optimal combination of mechanisms to reinforce their effectiveness is context-specific.

## 1 Background

Quality is a basic tenet of services delivery. It is also central to universal health coverage (UHC) as poor quality, independent of access, can be a barrier to UHC [1]. Sustainable Development Goal 3 has called attention to this critical link, as the attainment of health targets demands both the expansion of access to basic health services and enhanced quality; without the latter UHC will prove largely ‘an empty vessel’ [2]. Recent evidence on the magnitude and cost of deficits in healthcare quality, including deaths caused by adverse events in hospitals, high levels of excess and inappropriate care and unnecessary prescribing contributing to antimicrobial resistance among other public health concerns, have served to underscore the urgency of comprehensive health system efforts to improve quality alongside access [2].

It is clear the global health community is awakening to this challenge. In 2018, three flagship reports put a spotlight on the link between quality of care and global health [[2], [3], [4]]. These reports have presented the components for national health sector quality plans and policies [3], developed new approaches to define, measure and improve quality of care [4], and quantified the state of quality of care globally as well as the implications of current quality gaps [2]. Taken together with other recent studies – including a systematic review on the effectiveness of strategies to improve the performance of health care providers [5] and study on the use of quality strategies in the context of European health systems [6] – it is clear there is a critical mass of evidence and know-how to take concerted action. These studies build on earlier work on topics such as quality of care policies [[7], [8], [9], [10], [11]], strategies for quality improvement [[12], [13], [14], [15]] and the development of frameworks for monitoring and evaluating quality of care [[16], [17], [18]].

Despite the growing literature base and understanding of quality of care and its mechanisms – as the actionable interventions, strategies or tools to improve quality of care – thoughtful study on their *use* remains underdeveloped. Activating quality of care mechanisms is the responsibility of real-world health system stewards in their role to govern the health system and redress system limitations. The dilemma stewards face, however, is knowing which quality of care mechanisms will have the greatest influence on the health system and when to use them [10]. That is, in order to be ‘useful’ quality of care mechanisms should be embedded within the processes of governing and serve the intended target area of improvement in alignment with other quality of care mechanisms.

In the absence of guidance on how to optimize the use of quality of care mechanisms, popularized mechanisms like pay-for-performance, audit and feedback and clinical protocols and guidelines, are often pursued in practice as individual, catch-all quality enablers. The consequence is the inefficient and ineffective use of mechanisms to improve quality of care and overall health outcomes. For example, mechanisms like professional certification, clinical protocols or facility accreditation are typically activated for ensuring quality standards for the health workforce, clinical practice and facilities, respectively. However, stewards must also create enabling conditions for the system’s actors to translate policies and standards into action while working towards improving health outcomes and well-being. This process requires the use of different mechanisms, such as clinical supervision, quality improvement teams and facility inspections. Further to this, other mechanisms are also needed to assess performance and review the extent to which the original goals of improving health outcomes and well-being have been met. This process relies on the of mechanisms such as patient reported outcome measures, internal or external benchmarking and public reporting. It is this information that fuels learning and overall quality of care improvement by way of feeding back to inform future priorities. Importantly, this cycle applies to each component that makes up the health system – from the inputs such as the workforce and facilities, to the care provided such as clinical and emergency services and the intended users, as the public and patients.

Key to a practical, use-oriented approach to steering the activation of quality of care mechanisms, is an appreciation for the what, when and why: what mechanisms are available for use and have proven to be effective; when along the core processes or sub-functions of governance should they be activated and why – what is their primary focus or target area of the health system which also stands to gain the most from their use. Consultations with countries in the European context coordinated by the World Health Organization (WHO) have affirmed the absence, yet importance, of a taxonomy that addresses these considerations for use and maps policy options towards improved accountability for quality of care [19]. For health system strengthening at pace with health for all targets, frameworks to support evidence-informed decision-making and to catalyse the use of quality of care know-how are urgently needed.

## 2 Purpose and rationale

In this review, we adopt the perspective of health system stewards and set out with the aim to increase the optimal use of quality of care mechanisms through a policy-relevant and practical approach to health systems strengthening. To do so, we defined three specific objectives:

1. to catalogue quality of care mechanisms from systematic reviews and grey literature;
2. to plot quality of care mechanisms in a framework crossing the concepts of governance and its subfunctions and health systems thinking; and
3. to review best-available evidence on the effectiveness of the mechanisms identified.

To increase the actionability of the framework underpinning these objectives, we reasoned along two dimensions. The first, we refer to as ***governance sub-functions***. We adopt the approach to governance used in Smith et al. 2012 [20] and elaborated from WHO 2000 [21] and Travis et al. 2002 [22] where governance is described by its tasks “of defining the vision and direction of health policy, exerting influence through regulation and advocacy, and collecting and using information” [21].We refer to these tasks (activities, processes) as sub-functions [23] – functions subsidiary to the function of governance itself. Drawing from the literature [24], these processes are described as follows.

Setting priorities and standards refers to the highly political process involving advocacy and negotiation to set priorities and generate policies related to specific goals. To do so, mechanisms aim to convene stakeholders and formulate priorities into policies and standards. Organizing and monitoring action depicts the transition of policy into implementation by way of influencing the performance of multiple actors in order to steer the system towards set goals. Mechanisms in this sub-function aim to equip actors with the tools and resources to align their performance with priorities and standards. Further to implementation is the sub-function of assuring improvements in health outcomes and wellbeing. It depicts the task of generating and using information for learning based on the overall health outcomes associated with practitioners, organizations and the entire health system. Mechanisms in this sub-function aim to create actionable performance intelligence. The further feedback of these findings to set future priorities is the essence of the ‘steering’ function of stewards and has been described as inherent to the successful governance of health systems [20].

The second dimension we explore applies health systems thinking. We refer to it as a recent study describes as ***targets of quality of care mechanisms*** [20]. All quality of care mechanisms can be considered to be guided by the pursuit of better health outcomes. Nonetheless, their unit of focus or target varies. Systems thinking acknowledges the individual components of health systems to reveal their unique characteristics [25]. We apply a more delineated approach to the traditional ‘building blocks’ to capture at least three main targets of quality of care mechanisms: the public, as patients and the general public; services delivery, including clinical practice, emergency medicine, laboratory services, management of services; and system resources, including the health workforce, pharmaceuticals, health facilities and health information systems. The financing function, used by stewards to steer the system, is viewed as a governance enabler rather than an a *target* itself for quality improvement.

**Fig. 1:**
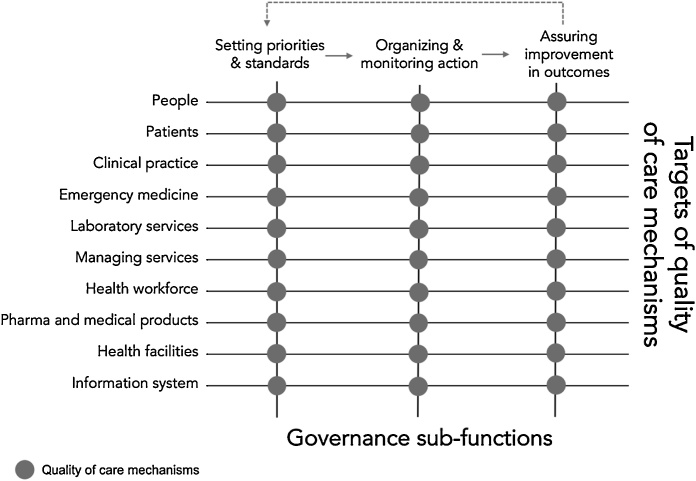
Framework applied to map the use of quality of care mechanisms by health system stewards.

Source: authors’ own.

We used the resulting matrix combining the sub-functions of governance and targets of quality of care mechanisms as the framework for our review (Fig. 1).

## 3 Methods

This work applies the methods of a scoping review [[26], [27], [28]] and was completed in three stages: (1) identifying quality of care mechanisms in systematic reviews and grey literature (2); assessing evidence on the effectiveness of quality of care mechanisms identified by reviewing findings of systematic reviews; and (3) plotting mechanism in the framework defined.

The work was conceived in April 2017 at a meeting organized by the WHO European Centre for Primary Health Care in Almaty, Kazakhstan. The two-day meeting brought together country representatives and quality of care experts from Europe and central Asia. Participants presented and discussed the use of quality of care mechanisms in their countries and respective areas of work. A consolidated list of quality of care mechanisms was presented at the closing of the meeting. Participants concluded the importance of developing a practical framework to improve the use of mechanisms and accountability for quality of care in practice [25].

### 3.1 Quality of care mechanisms defined

For the purpose of this paper, we define quality of care mechanisms (interventions, strategies or tools) as actionable interventions aimed at reducing quality deficiencies in health systems [29]. The primary aim of a quality of care mechanism is to improve one or more of the six dimensions of quality – safe, effective, patient-centred, timely, efficient and equitable care [30]. Quality of care mechanisms form the arsenal of resources that system stewards can mobilize in their effort to steer the system and bring alignment among actors working towards system goals [24].

Governance mechanisms are also needed to catalyse or enable the conditions for implementation and to hold actors accountable. These mechanisms are characterized in the context of this review as those used to create enabling system conditions to perform and achieve desired system goals. Evidence on the effectiveness of governance mechanisms identified in the literature reviewed has been consolidated. The full range and properties of these mechanisms has been explored elsewhere [23,31,32].

### 3.2 Process and sources of evidence

The processes of identifying mechanisms, mapping and reviewing evidence for each are described as follows.

#### 3.2.1 Stage one: identifying quality of care mechanisms

**Fig. 2:**
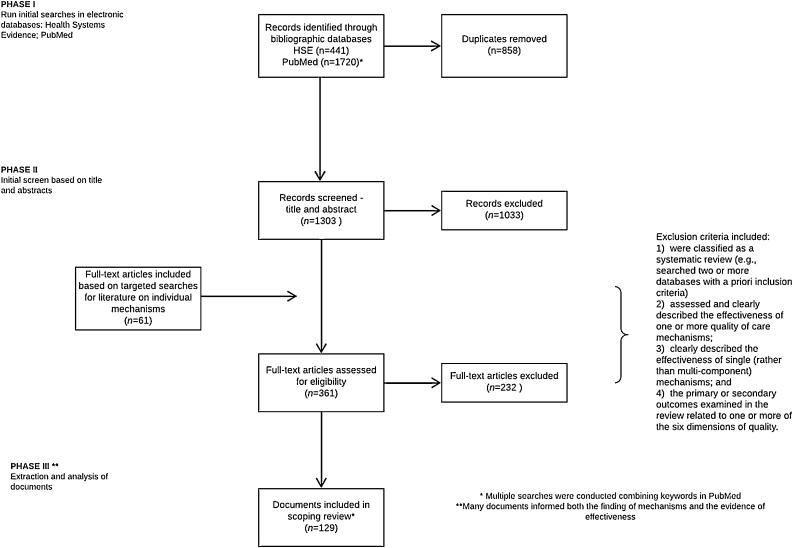
PRISMA diagram.

Building on the outcomes of the meeting in April 2017, an initial search to identify quality of care mechanisms was undertaken between June and September 2018. Primary searches were run in Health Systems Evidence, with targeted follow-up searches conducted in PubMed. The following MeSH terms were searched to identify a first set of systematic reviews: “quality of health care” “quality assurance” “quality improvement”. In Health Systems Evidence, filters were applied for “governance arrangements” “systematic review of effects” “systematic reviews addressing other questions” and “overviews of systematic reviews”. The search was also limited to reviews published in the past fifteen years. In PubMed, we applied filters for the same fifteen-year timeframe as well as adding “systematic review” to the MeSH terms above. The search strategy is documented in a PRISMA diagram (Fig. 2). In addition to systematic reviews, we reviewed 30 relevant reports from international organizations, such as the European Union, Organisation for Economic Co-operation and Development (OECD) and WHO. The reports were identified based on the knowledge and professional experience of one of the authors (JET). All reports and reviews had to be available in English to be considered. Supplementary file 1 details the full search strategy.

The assessment of systematic reviews to be included in the evidence summary was conducted by one author (KW), as is consistent with the methodology of a scoping review. Systematic reviews were included if they met the following pre-determined criteria: (1)]were classified as a systematic review (e.g. searched two or more databases with *a priori* inclusion criteria); (2)^2^] assessed and clearly described the effectiveness of one or more quality of care mechanism (3); clearly described the effectiveness of single (rather than multi-component) mechanisms; and (4) the primary or secondary outcomes examined in the review related to one or more of the six dimensions of quality [30]. Systematic reviews and relevant reports were used to identify quality improvement mechanisms.

All three authors reviewed the list to determine which mechanisms to include. In order to confirm the uniqueness of each mechanism, a glossary of terms was developed drawing from the literature reviewed (Supplementary file 2). From this, a consolidated, yet dynamic, list of quality of care mechanisms was developed. The list was further adjusted through the stages that follow.

#### 3.2.2 Stage two: mapping quality of care mechanisms

As a next stage, all three authors independently plotted the mechanisms in a preliminary framework. The full range of mechanisms were reviewed in alpha order and assigned an affiliation along the two dimensions (governance sub-functions and targets of quality of care mechanisms). The glossary of terms was the only file consulted in the process (Supplementary file 2). All mappings were consolidated. If there was disagreement for either dimension (approximately 30 %), these mechanisms were discussed in a joint meeting of the authors. Based on this discussion, mechanisms which could apply to more than one cell were marked as such. Definitions that required further specification were revisited in the original source.

The preliminary approach and mapping of quality of care mechanisms were further validated with decision-makers in four countries of the WHO European Region. This validation was conducted through country-specific workshops and key informant interviews between 2017–2018. Each country exercise is reported on as individual country studies: Belarus (2018) [33], Georgia (2017) [34], Kyrgyzstan (2018) [35] and North Macedonia (2018) [36]. In Belarus and Kyrgyzstan, a one-day workshop was organized with approximately 20–30 representatives of key quality actors including the ministry of health, health insurance fund, health care inspectorate and professional associations. In Georgia and North Macedonia, semi-structured interviews with representatives of equivalent profiles were conducted.

Participants and informants were asked to consider for each quality of care mechanism the following: its use in the current system, the actors responsible for the mechanism, and status of use (number of years, regulatory framework, context). Participants were also asked to consider if there were mechanisms missed in the listed set presented, the clarity of definitions provided, and accuracy of the mapping. All comments and discussion points were recorded and resulted in further adjustments to definitions. In a final stage of mapping, the authors further reviewed the clustering and definitions of the framework’s dimensions and conducted a full review of mechanisms on both axes. Further adjustments to the framework were made to fully adopt a steward’s perspective and their tasks to govern the system. Mechanisms may apply to more than one cell, however, to avoid repetition the primary target and contribution to a governance sub-function was prioritized.

#### 3.2.3 Stage three: reviewing evidence on effectiveness

Findings from systematic reviews found in the initial search were extracted using a set template to extract the following information: title of the review; date of last literature search; quality rating (Supplementary file 3: AMSTAR (quality) rating applied); quality mechanisms studied in the systematic review; and key findings on the effectiveness. This process was conducted by one author (KW). Additional targeted searches were then run for each mechanism for which no systematic review was found in the initial searches. Searches were run in Health Systems Evidence and PubMed using the name of the mechanism (and any relevant synonyms) as keywords. These were conducted on an ad-hoc basis up to November 2018 and resulted in the inclusion of an additional 60 systematic reviews. The data extraction for all included systematic reviews can be found in Supplementary file 4 on the effectiveness of quality of care mechanisms reviewed.

Data extraction was then reviewed to determine which mechanisms had sufficient evidence to either support or discount the effectiveness of the mechanism on one or more of the six dimensions of quality. AMSTAR scores were used to support this process in assessing the quality of the review. Table 1 provides details on how the level of evidence was assigned.

**Table 1:** Types of evidence statements and the level of evidence required to support the statement.

Evidence statement	Level of evidence required
Sufficient review-level evidence to either support or discount the effectiveness of the mechanism on quality	• Clear and consistent message coming from included high or medium-quality reviews. • Reviews included methodologically robust studies.
Tentative review-level evidence to either support or discount the effectiveness of the mechanism on quality	• A tentative statement from most of the included reviews. • Consistent evidence from a small number of reviews containing a small number of studies (of varying quality). • Conflicting (or mixed) evidence from one or more reviews included, with the stronger evidence weighted towards one side.
Insufficient review-level evidence to either support or discount the effectiveness of the mechanism on quality	• A statement of insufficient evidence from an included review. • Conflicting (or mixed) evidence from one or more reviews included. • No reviews on the mechanism, possibly due to a lack of robust primary studies available.

## 4 Findings

We conducted multiple searches in Health Systems Evidence and in PubMed resulting in 2086 records however, the searches had a significant number of duplicates (n = 783). After removing duplicates, there were 1303 results reviewed for title and abstract inclusion. Ultimately, 129 documents informed both the mapping of the mechanisms and evidence on effectiveness.

### 4.1 Identifying and mapping quality of care mechanisms

In total, we identified 130 quality of care mechanisms. What constitutes a quality of care mechanism in the literature and initial input from country representatives and experts varied considerably and were referred to as tools, strategies or interventions. In Table 2 all quality of care mechanisms identified are mapped to the dimensions of governance sub-functions on one axis and the target of the mechanism along the other. The table can be read from left to right to examine how the mechanism appears across the processes of setting priorities and standards, organizing for action and assuring health outcomes and wellbeing improvements. It can also be read from top to bottom to examine how the mechanisms map across the target areas. Those mechanisms with sufficient evidence to support their effectiveness in improving quality of care are bolded.

**Table 2:** Mapping of quality of care mechanisms identified.

Targets of quality of care mechanisms	Governance sub-functions		
	Setting priorities and standards *Process of setting priorities and translating* *goals into required standards.*	Organizing and monitoring action *Supporting implementation by providing* *actors the tools and resources to align* *their performance with priorities.*	Assuring improvement in outcomes *The process of leveraging data on the* *outcomes of care to assure improvements.*
People *Mechanisms aimed to ensure the public are supported to meaningfully take control of their health and health services.*	Citizens’ panels (juries) [[38], [39], [40]] Consumer association (groups) [38,39] Health service ombudsperson [41] Participation of community representatives in decision-making [38,42,43]	Consumer directed information [44,46,47,48] Consumer watchdog committee	–
Patients *Mechanisms aimed to engage patients to actively take part in their own health and health services.*	Patient associations (groups) Patient bill of rights (patient charter) Shared decision-making (strategy, protocol or standard) [45,49]	Automated alerts and reminders for patients [[50], [51], [52], [53]] Patient complaint systems Patient decision-aids (supports) [38,45,53] Patient education [50,51,[54], [55], [56], [57], [58]] Patient pathways (care pathway, care map) [53,59] Peer support groups (peer-to-peer supports) [60,61] Self-management [[50], [51], [52],^62^,^63^]	Patient reported outcome surveys [64,65] Patient reported experience surveys Patient satisfaction surveys [66] Patient feedback system [67]
Clinical practice *Mechanisms aimed to improve clinical practice.*	Clinical practice standards [29,68,69] **Clinical protocols and guidelines** [44,53,[70], [71], [72], [73], [74]] Disease registries	**Audit and feedback** [44,[53], [54], [55],^71^,[75], [76], [77], [78]] **Clinical decision support systems (incl. alerts and reminders)** [[53], [54], [55],^57^,^70^,^71^,^77^,[79], [80], [81]] Computerized diagnostics [69] Delayed prescribing [48] Discharge planning [82] Facilitated relay of clinical data to providers [29,50,51] Structured clinical vignettes Structured medication review/structured medication reconciliation [70,79,83,84] Clinical checklists [3,85]	Critical Incident Report/Adverse event reporting [29,86] Patient safety reporting system Morbidity and mortality reviews [87] ‘Never event’ reporting
Emergency medicine [ambulance) *Mechanisms aimed to ensure responsive emergency medicine services.*	Dispatch protocols [88] Standardized handover forms [89] **Equipment checklist** [88]	Care pathways including transfer pathways [88] Computerized decision support systems (incl. triage) [88] Dosing/code cards [90] Pre-admission patient data sharing [88,91]	Critical incident reporting [88,90]
Laboratory services *Mechanisms aimed to enhance laboratory services.*	Accreditation [92] Certification [92] International laboratory standards [92] Internationally recognized labels [92] Licensure [92] **Patient identifiers and sample identifiers** [93] Sample registry [92] Standard purchasing or procurement process [92] Standardized test request forms [92] Standard operating procedures	Circulation pathways [92] Equipment inventory list [92] Equipment maintenance logs [92] Equipment validation and function checks [92] Laboratory safety audits [92] Plan-Do-Study-Act cycles [92] Risk assessment [92] Computerized inventory management [[94], [95], [96], [97], [98]]	External quality assessments [92] Laboratory quality indicators [99]
Management *Mechanisms aimed to ensure quality managerial processes.*	Facility performance agreements [57]	Failure Modes and Effects Analysis [29] **Quality improvement collaborative across facilities** [57,100,101] Plan-Do-Study-Act [29,102] Quality improvement teams (quality circles) [73,76,103] Root Cause Analysis [104]	Benchmarking [57] External benchmarking [105] Facility performance indicators Internal benchmarking Quality service report cards
Health workforce *Mechanisms aimed to ensure a competent health workforce.*	Accreditation of certifying bodies Accreditation of training schools [106] Co-regulation [44] Health professional registry List (core set) of professional competencies Professional association (bodies, councils, chambers) Professional certification Professional licensing [3] Professional re-validation Professional self-regulation [44,107] **Simulation based training (including standardized patients)** [70,108,109] **Training and education** [3,44,48,49,55,69,70,75,82,103,110,111]	Clinical observation [112,113] **Clinical supervision**[44,49,71,107,[113], [114], [115]] **Continuous medical education** [49,53,116] Objective Structured Clinical Examinations [117] Peer-review teams/committees Professional re-certification [68] Task-shifting [114] Educational outreach [29,77,80] **Team changes [**[50], [51], [52],^70^,^111^,^114^]	Multi-source feedback assessment (360° assessment) [118] Public reporting on health professionals [57,[119], [120], [121], [122], [123]] Report cards of health professionals [44,54]
Pharmaceuticals and medical products *Mechanisms aimed to promote the use of pharmaceuticals and medical products.*	Barcoding of pharmaceuticals [124] Bulk purchasing [56] Essential Medicines List [3] Health Technology Assessment [125] Medicines formulary [55,56] Medicines registry Price controls Regulation of market entry (or market access) [56] Medicine authentication system [126,127] Standardized procurement processes [126] Medicines appearance checklists [126] Pre-approval inspection or mandatory inspection of new pharmaceuticals	**Computerized prescriber order entry** [55,70,79,128] Pharmacy and treatment committees Supply chain management [129] Audit of procurement processes Pharmacovigilance (system, centres or committees) [126,127]	–
Health facilities *Mechanisms aimed to ensure adequate health facilities.*	Changing facilities physical structures [103] Facility-based safety protocols [3] Facility accreditation [44,53,68,76,106,114,[130], [131], [132]] Facility certification Facility standards [133] Permits/permitting	Facility inspections [134]	Public reporting on performance by facilities [53,119,120,122,123]
Information systems *Mechanisms aimed to ensure optimal health information systems.*	Data protection and confidentiality protocols **Electronic health records** [57,135] Electronic patient registry [50,51] Unique patient identifier	–	–

Note: mechanisms are listed in alphabetical order by cell. Bolded terms denote mechanisms with sufficient evidence for their effectiveness on improving quality of care in the literature reviewed. Quality of care mechanisms without a reference were identified based on the insights and first-hand experiences of workshop participants.

### 4.2 Examining the evidence

A total of 129 overviews and systematic reviews were included in the review of evidence on the effectiveness of quality of care mechanisms identified. The quality of the reviews varied according to the AMSTAR scoring applied. Thirteen mechanisms were found to have a sufficient amount of evidence to support their effectiveness on improving one or more dimensions of quality. The key findings from each systematic review are presented in Supplementary file 4. These mechanisms include:

• audit and feedback;
• clinical decision support systems;
• clinical protocols and guidelines (when accompanied by other mechanisms);
• clinical supervision;
• computerized prescriber order entry;
• continuous medical education;
• electronic health records
• equipment checklists (in emergency medicine);
• patient identification and sample identifiers (in laboratories);
• quality improvement collaboratives across facilities;
• simulation based training (including standardized patients);
• team changes (including development of interdisciplinary teams); and
• training and education (when educational methods are mixed).

Another 33 mechanisms were found to have some evidence to support their effectiveness in improving quality. However, these mechanisms have been categorized as having “tentative review-level evidence” to support the effectiveness of the mechanism on improving quality of care. Many of these were categorized as such based on the findings from relatively few, low-quality systematic reviews (using AMSTAR rating tool) or a degree of mixed findings between included reviews (see Supplementary file 3 for additional information).

Table 3 maps the 128 quality of care mechanisms including those with sufficient evidence on their effectiveness. The clustering of mechanisms with sufficient evidence is similar to the distribution in Table 2. That is, the greatest number of mechanisms with sufficient evidence were found in relation to the governance sub-function of setting priorities and standards (6 of 58) and for organizing and monitoring action (7 of 50). By target areas, the greatest number of mechanisms with sufficient evidence on effectiveness were found in relation to the health workforce (5 of 24) and clinical practice (3 of 16).

**Table 3:** Range of quality of care mechanisms and evidence identified.

## 5 Discussion

Over time, the concept of quality of care has evolved from a notion of human error and negligence, with a resulting culture of blame, to the widely accepted understanding that quality is the combined result of system functions and requires a system-wide response [10,13,14,136,137]. Stewards have an integral role in creating conducive environments for such wide-reaching changes. We observe the following four implications of our findings for system stewards in this endeavour.

### 5.1 A new governance-oriented taxonomy for quality of care mechanisms

The mapping of quality of care mechanisms developed has a number of possible uses. First and foremost, it has the potential to support system stewards to identify the mechanisms that are currently in use in their system through a scan across the targeted areas, such the workforce, health facilities, pharmaceuticals, among the others explored. Further to this, aspects related to the scale of implementation and range of actors involved, for a mapping of existing accountability arrangements, could be pursued. The resulting overview is seen as an integral input to planning national quality of care policies or strategies.

When applied in countries, the taxonomy can aide system stewards to “get up on the balcony” – a metaphor that rightly captures the exercise of gaining an overview of efforts in motion [138]. This overview can ensure stewards are equipped to manoeuvre changes and create the institutional and enabling conditions across the sub-functions of governance that allow for synchronized efforts of the system’s actors. The validation process in countries served as a preliminary test of an approach to map quality of care mechanisms for this purpose. The taxonomy of mechanisms can also serve as an inventory of additional or alternative mechanisms to be assessed and activated based on strategic quality goals.

### 5.2 A paradigm shift towards a systems-focused and use-oriented approach to quality of care mechanisms

Our mapping confirmed there are unique mechanisms across the two dimensions explored. Nonetheless, there is a wide range of variability in the number of mechanisms by cells. We observe this range in patterns that reflect the development of health systems and quality of care priorities overtime [139,140]. For example, along the target areas of quality of care mechanisms, the greatest range of mechanisms and evidence of effectiveness correspond to long-established priorities of improving clinical practice, ensuring a competent workforce and promoting the responsible use of medicines (Table 3). We find a less diverse range of mechanisms for areas that have received recent yet increasing policy importance, such as empowering people, engaging patients, and managing services.

Similarly, there is clustering of the mechanisms along the sub-functions of governance. Specifically, the greatest number of mechanisms were found to align to setting priorities and standards (Table 3). We interpret this finding in connection to the tradition of prioritizing quality system inputs, in the logic of equipping services delivery with optimal resources, e.g. defining entry-to-practice licensure, certification or registration standards of health care practitioners towards a competent workforce. The second largest range of mechanisms were identified in the scope of organizing for action to translate policies into practice. This finding attests to the fact that priorities and standards alone do not safeguard quality of care. These mechanisms are the tools and resources that enable the capacity of actors to comply with established standards and include, for example: clinical supervision, continuous medical education or peer-review teams (health workforce); care pathways including transfers, and dosing/cards (emergency services); or pharmacy and treatment committees, audit of procurement processes or medicines appearance checklists (pharmaceuticals), among others.

Further to supporting the implementation of priorities and standards are those mechanisms used to generate information on health outcomes. In doing so, these mechanisms facilitate learning and setting future priorities and standards based on identified areas for further improvement. Mechanisms identified include for example, patient-reported experience and outcome surveys, public reporting and report cards of health professionals. The smaller range of mechanisms mapped to this sub-function is found consistent with the field. That is, while the past decade has seen increasing attention to the collection of performance data, this does not immediately translate to the use of collected data for decision-making purposes [6,20].

We hypothesize countries with more developed or mature health systems activate a range of mechanisms along both dimensions of the framework applied. As countries work to improve quality of care and health outcomes, the two-dimensional approach may offer guidance to signal where there are gaps in target areas, and where – along the processes of setting priorities and standards, organizing and monitoring for action and assuring improvement including the important feedback of findings to inform future priorities – there is needed investment for the strategic use of mechanisms. A preliminary exploration of the use of mechanisms was pursued through the country validation process. This confirmed the diverse use of mechanisms as well as the wide range of actors involved and varied scale of implementation, e.g. some mechanisms implemented regionally or on a pilot basis. A comparative study exploring mechanisms in practice across countries may offer further insights into the development of quality of care along the continuum foreseen.

### 5.3 The evidence-base for mechanisms as a resource for decision-making

Further to a focus on the specific *use* of mechanisms (regarding their respective targets and alignment to the sub-functions of governance), evidence on their effectiveness is a necessary input for the decision-making of stewards. Research evidence can play an important role in this regard. However, a steward’s need for evidence (i.e. whether to clarify options, understand the benefits or to appreciate implementation considerations) and the relevant type of evidence should be considered as context specific [141].

The evidence review conducted found less than 10 percent of the mechanisms identified have a sufficient amount of evidence to support their effectiveness on improving one or more dimensions of quality. This can be attributed to many mechanisms having been identified in grey literature and the fact that they draw from the list of mechanisms initially generated with country representatives and quality of care experts based on first-hand experiences. The lack of available evidence should not be taken as a judgement on the relative effectiveness of other mechanisms and rather may be representative of the absence of systematic reviews. The lack of evidence at the review-level may also be a result of heterogeneity in primary studies, complicating the ability of researchers to conduct systematic reviews and meta-analyses. It may also be reflective of a lack of research on the effectiveness of single mechanisms (rather than the implementation of a range of complimentary mechanisms) or the broad range of contexts in which these mechanisms are being implemented and studied.

This finding has a number of implications for decision-makers. It means the quality of care mechanisms identified should be considered as inventory rather than a checklist. Moreover, the evidence presented in the review should be considered as a starting point for stewards and can be complemented by local evidence on the benefits, harms and costs of the mechanisms above as well as by the expert opinions of local stakeholders.

There remain significant questions regarding the effectiveness of one component intervention compared to multi-component interventions. While this review only examined the effectiveness of individual mechanisms, findings from a recent high-quality systematic review revealed that the effectiveness of multicomponent interventions is nuanced [5]. Specifically, the review found that the effectiveness of mechanisms was unrelated to the number of components included in the intervention. Therefore, the number of mechanisms combined does not directly improve their effectiveness. Rather, informed decisions on which mechanisms to combine – given the local context of the health system and available evidence – is critical [5].

### 5.4 Enabling governance conditions

In the search for mechanisms to support improvements in the quality of care, the importance of their implementation in the *right* environment was a consistent theme across the literature. The *right* environment was considered to be one in which the governance and financing arrangements of the health system were aligned with the aims of the quality of care mechanisms activated. This finding underscores the importance of ensuring that accountability arrangements are conducive to the implementation of quality of care mechanisms across health system actors and that financial arrangements reward their use and disincentivize low quality of care. The below evidence summarizes the findings from the systematic reviews on the enabling governance conditions for creating the *right* environment to optimize quality of care mechanisms.

#### 5.4.1 Accountability arrangements

Governance arrangements include changes in the rules or processes that determine authority and accountability for health policies, organizations, commercial products and health professionals, as well as the involvement of stakeholders [121]. Changes to these arrangements usually include adjusting the mandate, accountability and participation of actors to better support the implementation of mechanisms. We found evidence on three accompanying governance arrangements that support alignment with a quality of care agenda and ease the introduction and adoption of quality of care mechanisms. The first was the delegation of decision-making which was found to have mixed effects with one review reporting greater responsiveness to local conditions while another warned that this could be detrimental if greater accountability does not accompany the change [142,143]. The second was the inclusion of diverse stakeholders in policy and organizational decisions, finding that their participation can support improved decisions so long as they are supported with open communication and a non-hierarchical environment [121,142,144]. Finally, community and public engagement was found, when implemented correctly, to improve quality and outcomes as well as potentially helping to support sustained system changes when community buy-in is achieved [142,145].

#### 5.4.3 Financing arrangements

At their simplest, financing arrangements are the ways in which funds flow through the health system. These arrangements can reward or disincentivize providers and individuals to do or behave in particular ways. Financing arrangements can have both a direct and an indirect impact on quality of care. We reviewed the evidence on the effectiveness of co-payments, vouchers, pay-for-performance and value-based purchasing.

With regards to co-payments the evidence generally found that they can be used to signal high-quality care from that lower quality. The reviews suggest that reducing cost-sharing for high-value services, while increasing the out-of-pocket share for low-value services can steer service provision. However, co-payments should be used sparingly given their potential to also reduce the use of effective and life-saving treatments, notably among low-income groups [[146], [147], [148]].

A significant amount of evidence was found that related to pay-for-performance and value-based purchasing. While the literature found relatively few high-quality studies, there is significant evidence to show that both providers and patients respond to financial incentives [83,[149], [150], [151], [152], [153], [154], [155], [156], [157], [158], [159], [160], [161]]. Pay-for-performance schemes appear most effective for improving simple processes or structures of care but have no or largely unknown effects on health outcomes [149,[151], [152], [153],^157^]. The effects of these incentives appear to be relatively short-lived, with reviews reporting a return to baseline levels at the one year follow-up [153].

Reviews generally agree that pay-for-performance schemes tend to be more effective in ambulatory care than in specialized care and have led to larger effect sizes in reviews on preventive services, however one review notes that this finding may be overemphasized in the literature [152]. Mixed evidence was found for whether the magnitude of the incentive is associated with the effective size, with one review noting no association has been found while another reported that incentives are three times more likely to show a positive effect with larger incentives [152,153]. Most reviews noted that the effectiveness of pay-for-performance schemes are dependent on the context in which they are implemented and the way in which the incentive is designed, with one review noting seven key characteristics that influence the response of health professionals, including: payment rate, sufficiency of payment rate to cover the cost of services, timeliness of payment, payment schedule, performance requirements and accountability [150].

### 5.5 Limitations

This scoping review has five key limitations that should be acknowledged. The first is that the list of mechanisms is not exhaustive. While significant efforts have been undertaken to ensure a comprehensive list was developed, including undertaking three rounds of searches in two databases as well as reviewing recent reports from international organizations including the OECD, World Bank and WHO, it is unlikely that all mechanisms have been represented. Second, the mapping of each mechanism along the two dimensions of the framework applied was based on how it was described in the literature and on the expert opinion of the authors. Further validation is therefore needed. Third, the classification of mechanisms along both dimensions were at times blurred; in many instances, mechanisms could be used to improve quality of care of multiple targets. To resolve this issue, only the primary aim of each mechanisms has been captured in assigning each mechanism a specific cell. Fourth, there are limitations in the evidence included in the review. For the purpose of mapping individual mechanisms, only evidence examining the effects of single mechanisms was included. However, given that mechanisms are often implemented in parallel to each other (for example, the implementation of clinical guidelines with audit and feedback) there is significantly more evidence available for multi-component interventions than was included in this scoping review. Moreover, there is a significant amount of heterogeneity in primary studies examining the effectiveness of quality of care mechanisms complicating the development of systematic reviews. The decision to focus exclusively on systematic reviews was made both because of the reduction in bias of findings and for the timeliness of this review (which was conducted in six months). However, this decision did mean compromising on the inclusion of findings from primary studies. Finally, despite the focus on empowering people, no explicit searches were made for mechanisms involving families and carers.

## 6 Conclusion

The link between quality and the attainment of global UHC targets has driven attention to the fundamental importance of enhancing quality of care alongside increasing access to services. To this end, health system stewards have a paramount role in making progress. In this review, we adopted the perspective of system stewards and explored the range and evidence base for quality of care mechanisms. The resulting taxonomy of quality of care mechanisms can serve as a tool to scan the use of mechanisms in a given system, to identify gaps and to provide options for prioritizing action.

## Ethics approval and consent to participate

Not applicable.

## Consent for publication

Not applicable.

## Availability of data and material

All data generated or analysed during this review are included in this published article as a supplementary file.

## Funding

This work was carried out with the financial support of the Government of Kazakhstan through the WHO European Centre for Primary Health Care in Almaty, Kazakhstan.

## Authors’ contributions

Conception: JET; (II) Literature review and data extraction: KW; (III) Analysis: all; (IV) Drafting: EB, KW; (V) Critical review: JET; (VI) Revisions: all.

## Declaration of Competing Interest

The authors declare that they have no competing interests.
