# Supplementary material for: Review of 128 quality of care mechanisms: A framework and mapping for health system stewards
Source: Health Policy. 2020 Jan;124(1):12–24. doi: 10.1016/j.healthpol.2019.11.006 (PMC6946442; doi:10.1016/j.healthpol.2019.11.006)
Supplement: Supplementary file 1 [file mmc1.docx]

**Appendices**

**Supplementary file 1**

Title: Search strategy

Description: Search strategy used for grey literature and systematic reviews to identify quality of care mechanisms in Health Systems Evidence and PubMed.

**Supplementary file 2**

Title: Definitions of quality of care mechanisms identified

Description: Glossary of terms for quality of care mechanisms identified through the review used to differentiate mechanisms and determine their alignment in the framework. Glossary terms are listed in alphabetical order by mechanism.

**Supplementary file 3**

Title: AMSTAR (quality) rating applied

Description: Instrument used to assess the methodological quality of systematic reviews.

**Supplementary file 4**

Title: Summary of evidence on effectiveness of quality of care mechanisms

Description: Synthesis of the evidence found on the effectiveness of individual mechanisms on improving quality of care

**Supplementary file 1: Search strategy**

The first stage of the scoping review focused on developing a list of quality of care mechanisms. To do so, we undertook eight searches using the following search strategies in Health Systems Evidence:

The following MeSH terms were used to identify the first set of systematic reviews: “quality of health care” “quality assurance” “quality improvement”. In Health Systems Evidence, filters were used for “governance arrangements” “systematic review of effects” “systematic reviews addressing other questions” and “overviews of systematic reviews” were used, as well as limiting the reviews to the past fifteen years. In PubMed, we applied filters for the same fifteen-year timeframe as well as adding “systematic review” to the MeSH terms above.

Initial searches were conducted in June and July of 2018. Additional searches were conducted in PubMed using quality assurance as a Major MESH term. Targeted searches were run for each of the building blocks where limited reviews were found in Health Systems Evidence. We conducted three targeted searches using the following search strategies: 1) (quality assurance) AND (health workforce OR health human resources OR health professionals) AND (effectiveness); 2) (quality assurance) AND (information technology OR information systems) AND (effectiveness); 3) (quality assurance) AND (emergency medicine OR ambulance OR paramedic) AND (effectiveness). These searches were conducted between October and November 2018. Finally, ad hoc searches were run in Health Systems Evidence and PubMed for mechanisms where no systematic reviews were identified. Search strategies for these final additions combined quality assurance with the identified mechanism.

**Supplementary file 2: Definitions of quality of care mechanisms identified**

Glossary of terms for quality of care mechanisms identified through the review used to differentiate mechanisms and determine their alignment in the framework. Glossary terms are listed in alphabetical order by mechanism.

1. **Accreditation (labs)** is the process by which an authorized body, usually non-governmental organization, assesses and recognizes a laboratory as achieving pre-determined and published standards, demonstrated through an independent, external, periodic, on-site peer assessment of that organization's level of performance. Accreditation standards are usually regarded as optimal and achievable and are designed to encourage continuous improvement efforts within the accredited organizations.
2. **Accreditation of certifying bodies** (of health professionals) is the certification of an organization, often a nongovernmental agency, that grants it the authority to award time-limited recognition to an individual verifying that he/she has met predetermined standardized criteria to practice medicine. Certification has traditionally been the activity of professional associations. Through accreditation, organizations (e.g. professional associations) can be mandated the function of self-regulating.
3. **Accreditation of training schools** is the certification of the suitability of medical education programmes and the competence of the school in the delivery of medical education that ensures the competencies of future health professionals. Accreditation of training schools is often carried out by national governments or by agencies receiving their authority from national governments *(1)*.
4. **Audit and feedback (clinical)** refers to any summary of clinical performance of health care over a specified period of time aimed at providing information to health professionals to allow them to assess and adjust their performance *(2).*
5. **Audit of procurement processes** is a review of different contracts and contracting processes to determine the adequacy, completeness, and efficacy of obtaining a health product or medicine.
6. **Automated alerts and reminders for patients** is a mechanism to support self-management by alerting and reminding patients to accurately manage their treatment regimen in their daily routines (e.g. prompt to take medicines, check blood pressure, etc.). Automated alerts and reminders can facilitate shared accountability for disease management between health professionals and the patient *(3)*.
7. **Barcoding of pharmaceuticals** refers to applying a standard barcode to the inner labels (or the unit of use packaging) of products to ensure the right medication, at the right time, in the right dose and by the right route of administration is being provided to a patient *(4).*
8. **Benchmarking** is different than performance compared to standards whereby benchmarking applies data e.g. risk-adjusted 30-day rates for death, to determine the top 10% of facilities and benchmark particular measures (e.g. waiting times) based on top performing facilities *(5)*. Benchmarking can be used both for improving the performance of service outputs or for improving specific health outcomes.
9. **Bulk purchasing** is purchasing done by one procurement office on behalf of a group of facilities, health systems or countries, group members agree to purchase certain drugs exclusively through the group *(6).*
10. **Care pathways including transfer pathways** is a complex intervention for the mutual decision-making and organisation of care processes for a patient during a well-defined period. Care pathways may include an explicit statement of the goals of care, facilitation of the communication between patients and care team members, coordination of care within a multidisciplinary team, and the documentation monitoring and evaluation of variances and outcomes *(7).*
11. **Certification (labs)** is the formal recognition of a laboratory’s capability to carry out specific tests and tasks in a manner that meets a predefined standard.
12. **Changing facilities physical structures** are any renovations or alterations made to a facility’s physical structure to enable improvements in care delivery *(8).*
13. **Circulation pathways** are used to identify where improvements in laboratory design may be needed in order to prevent or reduce risks of cross contamination. The review process follows the path of the sample as it moves through the laboratory during the pre-examination, examination and post-examination phase of testing *(9).*
14. **Citizens’ panels (juries)** are a mechanism for citizen engagement to bring together diverse representation, supported by a range of resources, to discuss issues of public concern through a structured deliberation process. Health-related citizens’ juries may discuss topics such as ethical issues in population health, priority setting, health policy, environmental health, community well-being *(10)*.
15. **Clinical checklists** is a tool designed to improve the safety and standardization of clinical practice, with a common example being the use of surgical safety checklists *(11).*
16. **Clinical decision support systems** refers to any system designed to improve clinical decision-making related to diagnostic or therapeutic processes such as informing the selection of medicines or diagnostic tests. Typically these systems responds to triggers or flags for specific diagnoses, medication choices or laboratory results and provides information or recommendations. Reminder systems or computerized order entry systems are often considered as types of clinical decision support systems or may be embedded in more complex systems *(12)*.
17. **Clinical observation** is a process by which a clinician in training observes a fully certified health professional to observe skills in practice.
18. **Clinical practice standards** refer to authoritative statements that articulate minimum, acceptable or excellent levels of performance in services delivery *(12)*.
19. **Clinical protocols and guidelines** refer to systematically developed, evidence-based recommendations that support health professionals and patients to make decisions about the most appropriate, efficient care in specific clinical circumstances *(13),(14),(15),(16).*
20. **Clinical supervision** refers to the provision of guidance in clinical practice for qualified health professionals by a more experienced professional. Clinical supervision is used as a professional development activity where the less experienced clinical can use the knowledge and experience of their supervisor to address gaps in their own knowledge or skills sets *(17)*.
21. **Co-regulation** refers to the governance structure when a government enters into a partnership arrangements with an industry body or a professional association to regulate entry into the profession *(18).*
22. **Computerized decision support systems (including triage)** refers to any system designed to improve clinical decision-making related to emergency medicine processes including the selection of any interim actions before the patient is brought to the hospital or choice of triaging of patients once at the emergency room.
23. **Computerized diagnostics (computer-aided diagnosis)** is the electronic process to model the signs and symptoms of patients to support the accurate final diagnosis of health professionals. Computerized diagnostic support systems can minimize errors and improve the accuracy of diagnosis through real-time algorithms.
24. **Computerized inventory management** (also referred to as computerized laboratory information system) is a computerized database which holds records of all samples and reagents within a laboratory to bring accuracy and accessibility to the flow of samples and data in the clinical laboratory *(9).*
25. **Computerized prescriber order entry** is the electronic ordering system for the selection, display, storage of drug histories as well as electronic transmission of drug orders to dispensing pharmacists and pharmacies.
26. **Consumer associations (groups)** include associations and organizations that represent the rights of consumers and advance their interests. Consumer associations (groups) may participate in processes of priority setting, health policy debates or as members to advisory boards and committees on topics related to consumer rights, entitlements, public advocacy, etc.
27. **Consumer directed information** refers to educational materials specifically designed for health consumers with the aim of improving health literacy about a particular health-related topic or condition.
28. **Consumer watchdog committee** are an organized public group responsible for monitoring practices against illegal or unethical conduct of health insurance companies, drug companies, other profit-driven and/or public health services authorities. Watchdog committees may contribute to public monitoring, to provide administrators with a critical view of progress and practices.
29. **Continuous (continuing) medical education** is a process of continuous learning by which medical professionals keep themselves updated through acquisitions of new knowledge, skills and attitudes to maintain professional competency, in-patient management, health services management and their own professional development *(19).*
30. **Critical Incident Reporting/adverse event reporting** refers to a no-blame reporting system whereby critical incidents, as unintended events that occur when health services are provided to an individual and result in a consequence that causes serious and undesired harm. Critical incident reporting does not place blame on an individual. The process does not replace other disciplinary investigations such as reviews by employers, complaints to professional regulatory bodies or civil law suits. Critical incident reporting complements these processes.
31. **Critical incident reporting (emergencies)** refers to a no-blame reporting system whereby critical incidents, as unintended events that occur when health services are provided to an individual and result in a consequence that causes serious and undesired harm in the emergency department.
32. Data protection protocol (confidentiality protocols) are pre-defined standards concerning the handling of patient information, its use and exchanges to ensure that patient information is managed confidentially, and that legal, ethical and contractual obligations of confidentiality are disclosed and to be upheld.
33. **Delayed prescribing** is a technique whereby a prescription is issued by a health professional for use by the patient at a later date, if their symptoms do not improve. In parallel, patients are encouraged to self-manage their needs and provided supports and strategies to do so, accessing a prescription only if their condition does not improve after a specific period of time *(1).*
34. **Discharge planning** is the development of a personalised plan for each patient who is leaving hospital, with the aim of containing costs and improving outcomes. Discharge planning should ensure that patients leave hospital at an appropriate time in their care and that, with adequate notice, the provision of post-discharge services are organized *(20).*
35. **Disease registries** collects information over time on patients who are diagnosed with a particular disease or who receive particular treatments. Patient registries can serve scientific, clinical or policy purposes. Terms such as clinical registries, clinical data registries, disease registries and outcome registries can be used to describe this same process *(21).*
36. **Dispatch protocols** are standardized processes used by emergency call-takers to prioritize the arrival of medical services and to provide instructions to the caller before health professionals arrive.
37. **Dosing cards** refers to small cards that can be held on an emergency medicine professional to remind them of safe dosage amounts for critical medications.
38. **Educational outreach** is a face-to-face visit by a trained person to a health provider to their own setting for learning purposes. It may also be referred to as university-based educational detailing, public interest detailing, and academic detailing *(22).*
39. **Electronic health records** is a systematic electronic collection of health information about patients such as medical history, medication orders, vital signs, laboratory results, radiology reports and physician and nurse notes *(23).*
40. **Electronic patient registries** is an electronic collection – for one or more purposes – of standardized information about a group of patients who share a condition or experience *(24).*
41. **Equipment checklist** are continuously updated lists of the equipment including all medical supplies in contained in emergency medical vehicles.
42. **Equipment inventory list** is a list that is updated continuously to provide a correct look at the status of the equipment in the laboratory *(9).*
43. **Equipment maintenance logs** continuously updated list of any maintenance provided to all laboratory equipment.
44. **Equipment validation and function checks** is a process used to verify that equipment is working according to the manufacturer’s specifications. This should be done before using the instrument initially, then with the frequency recommended by the manufacturer. These checks should also be done following any repairs and could include activities such as monitoring of temperatures and checking the accuracy of calibrations *(9).*
45. **Essential Medicines List** is the prioritization of medicines that satisfy the health needs of a population and to which people should have access to at all times in sufficient amounts. Each country is encouraged to prepare an official essential medicines list that enables health authorities to optimize pharmaceutical resources *(25).*
46. **External benchmarking** is the comparison between a health professional or facility’s performance against a best practice set by an outside organization or agency as well as relative to other outside professionals or facilities. This mechanism can be used to evaluate either outputs or outcomes.
47. **External quality assessment (labs)** is used to describe a method that allows for comparison of a laboratory’s testing to a source outside the laboratory. This comparison can be made to the performance of a peer group or to a reference laboratory *(9).*
48. **Facilitated relay of clinical data to providers** is used to describe the transfer of clinical information collected directly from patients and relayed to the provider in instances where the data are not generally collected during a patient visit or using some format other than the existing local medical record system *(26).*
49. **Facility accreditation** is the process by which an authorized body, usually non-governmental organization, assesses and recognizes a facility as achieving pre-determined and published standards, demonstrated through an independent, external, periodic, on-site peer assessment of that organization's level of performance. Accreditation standards are usually regarded as optimal and achievable and are designed to encourage continuous improvement efforts within the accredited organizations. Accreditation is often a voluntary process in which organizations choose to participate*.*
50. **Facility certification** refers to a process by which an authorized body, either governmental or nongovernmental organization, evaluates and recognizes an organization/facility as meeting pre-determined requirements or criteria. It usually implies that the organization/facility has additional services, technology, or capacity beyond those found in similar organizations/facilities *(13),(27),(28).*
51. **Facility inspections** are investigations conducted by a qualified team (e.g. dedicated health inspectorate) to evaluate facilities against agreed upon standards. Inspections may be unannounced or occur as a result of a complaint. Inspections aim to protect the interests of the public and to identify violations to be addressed.
52. **Facility performance agreements (business plans, operational plans)** a detailed plan developed by a facility on a regular or semi-regular basis that provides a funding agency, local or national government with specifics such as an operating plan (including budgets) and performance indicators against which the facility can later be measured.
53. **Facility performance indicators** are measures by which to evaluate the success of a facility on a particular activity or service delivered and are designed to summarize information to suggest issues for performance management, quality improvement or further scrutiny by the facility *(29).*
54. **Facility standards** are a formalized list of key functions, activities, processes, structures and systems required for health facilities to be in a position to provide quality services. Facility standards can be determined by professional and regulatory bodies, health professionals, staff, patients and citizens. Facility standards can be accredited by the International Society for Quality in Health Care (ISQua) which certifies standards met internationally agreed upon principles and requirements *(30).*
55. **Facility-based safety protocols (or procedures)** are step-by-step instructions about how to mitigate or manage risk to patients’ and staff’s safety when providing care. Protocols or procedures will vary significantly based on the setting and type of care being provided.
56. **Failure Modes and Effect Analysis** is a method that attempts to identify all possible failures that can occur within a system and then determines what the effects of those errors would be. The analysis aims to identify recommended actions that would diminish the severity or occurrence of failures and to put in place mechanisms to improve error detection *(31)*.
57. **Health professional registry** refers to an authoritative source of health workforce information that can provide an accurate count of all health care personnel that either have worked or are currently working at a national or sub-national level, including in the private sector (where applicable) *(32).*
58. **Health service ombudsperson** is an official, often reporting to the government, who is appointed to protect the interests of the public and patients in relation to administration of and provision of health services. A health services ombudsperson can investigate complaints about health authorities, insurers and/or practitioners.
59. **Health Technology Assessment** is the systematic evaluation of the properties, effects or other impacts of health technologies. It is intended to inform decision-makers about health technologies and may measure the direct or indirect consequences of a given technology or treatment *(18).*
60. **Internal benchmarking** is the comparison between a health professional or facility’s performance against an internally set best practice *(33).*
61. **International laboratory standards** are standards or benchmarks set by a number of organizations for quality manufacturing and service industries to support quality management *(9).*
62. **Internationally recognized labels** are the classification of chemicals by types of hazard and proposes harmonized hazard communication elements, including labels and safety data sheets. They are used to ensure that information on physical hazards and toxicity from chemicals is clear to those handling, transporting and using these chemicals *(34).*
63. **Laboratory quality indicators** are the specific targets that are regularly examined using objective methods, in order to determine if the goals of compliance are being met. When developing quality indicators an organization should ensure the indicators are measurable, that the organization has the tools needed to accomplish the measurements, and the acceptable value before starting measurements (9).
64. **Laboratory safety audits** are assessments conducted by groups or agencies from inside or outside the laboratory to ensure the safe operation of equipment and testing of patient samples (9). .
65. **Licensure (laboratories)** is the process by which a governmental authority grants permission, usually following inspection against minimal standards, to a laboratory to operate.
66. **List (core set) of professional competencies** is a set of essential knowledge, skills and attitudes of health professionals that, together with existing and available resources, ensure safe and quality outcomes for patients and populations. Core competencies may relate to skills for patient advocacy, effective communication, teamwork, people-centred care, continuous learning *(35)*.
67. **Mandatory inspection of new pharmaceuticals (or pre-approval inspection)** refers to processes put in place before a new drug enters the market whereby a government agency or medicines regulatory body assesses whether the methods used in, and the facilities and controls used for, the manufacturing, processing, packing, and testing of the drug are found adequate to ensure and preserve its identity, quality and purity (36).
68. **Medicines formulary** a list of medications covered by a given insurance plan or stocked by a given facility along with information about the medication. Ideally formularies should match to national essential medicines lists to ensure alignment with nationally prioritized medicines.
69. **Medicines authentication system** is the use of unique alpha-numeric numbers for medicines that can be confirmed with the manufacturer (often through SMS or searched in information systems) to ensure its identity and quality *(37).*
70. **Medicines appearance checklist** is a tool designed to help health professionals carry out a visual inspection of medicines for signs of counterfeiting such as improper packaging, labelling or description of dosage *(38)*.
71. **Medicines registry** is a list of all medicines available for sale or previously available for sale in a given jurisdiction.
72. **Morbidity and mortality reviews** refers to an approach that provides members of a health services team the opportunity for peer review of adverse events, complications or mortality to reflect, learn and improve patient services *(39).*
73. **Multi-source feedback assessment** is increasingly used as a formative or summative assessment of health professionals’ competences, including clinical skills, personal communication and patient or client management. The assessment requires that health professionals complete a self-assessment of a given competency (using a pre-developed questionnaire) that is then examined by medical colleagues, nonmedical co-workers or patients to provide their own feedback *(40)*.
74. **‘Never event’ reporting** refers to adverse events that are unambiguous (clearly identified and measurable), serious (resulting in death or significant disability) and usually preventable. A classification of never events may include surgery or other invasive procedure performed on the wrong body part or wrong patient *(5)*.
75. **Objective Structured Clinical Examinations** is an evaluative tool to assess health professionals’ competencies in a clinical setting through direct observation. These examinations differs from other clinical exams by assessing competencies such as communication skills *(41)*.
76. **Participation of community representatives in decision-making** refers to mechanisms that support eliciting the views of the public in policy decisions regarding the health system, this may take shape at different levels including the creation of a patient or family council or inclusion of community members in the governance of health organizations.
77. **Patient associations (groups)** are organizations that provide insight and represent patient experiences as potential, current and past recipients of health services on general health topics or disease-specific areas *(35)*. Patient associations (groups) may have a formal role in processes of priority setting, health policy debate, trainings of patients, etc.
78. **Patient bill of rights (patient charter)** is a document, typically published by government, that affirms the rights and entitlements of consumers and patients with respect to health services. The document may also describe consumers’ or patients’ expectations for achieving a given standard of care.
79. **Patient complaint system** is a systematic and transparent process for receiving, investigating and resolving patients expressions of grievance or disputes with the care they received.
80. **Patient decision-aids (supports)** are tools that help patients, their families and carers to be informed and involved in decision-making by making explicit the options available and their outcomes, and by clarifying personal values. Patient decision aids are designed to complement, rather than replace, counselling from a health professionals *(42).*
81. **Patient education** refers to process by which health professionals and other health workers impact information to patients and their families to alter their health behaviour, improve their health status, or allow them to make informed decisions regarding their care.
82. **Patient feedback system** is a standardized process for collecting feedback from patients on their experiences with health services *(43)*. It may take different forms including in-person (e.g. in-depth interviews, focus groups), written (e.g. compliments and complaints forms, comment boxes), or electronic (e.g. tablets and mobile applications to provide instant feedback on or rate services using a scoring system).
83. **Patient identifiers and sample identifiers** are tags or barcodes used to keep track of the patient and their sample.
84. **Patient pathway (care pathway, care map)** refers to an aid (in additional clinical guidelines) that maps a patient’s journey through the health system. It plans for the management of patient care that sets goals for the patient and provides the sequence of interventions that health professionals should carry out in order to reach the desired goals in a given period of time *(19,30).*
85. **Patient reported experience surveys** are a means for patients to report on their experience of being treated (e.g. whether the treatment was properly explained, if they felt involved in decisions about their care) *(44)*.
86. **Patient reported outcome surveys** are a means for patients to report on the outcomes that matter to them (e.g. whether treatment reduced their pain, or if it helped them live more independently) *(44)*.
87. **Patient safety reporting system (adverse event reporting)** are voluntary reporting systems which rely on those involved in events to provide detailed information on the incident. It is a *passive* form of surveillance for near misses or unsafe conditions, in contrast to more *active* methods of surveillance such as direct observation or chart review *(5)*.
88. **Patient satisfaction surveys** are a systematic process to generating feedback from patients. Satisfaction surveys are generally considered more generalizable than individual feedback.
89. **Peer support groups (peer-to-peer support)** are patient-driven groups on specific topics that encourage individuals to be in direct control of managing their conditions through group work and mutual support allowing them to draw on each other’s experiences.
90. **Peer-review teams (committees, circles)** are small groups of health professionals based on voluntary participation and concerned with activities aimed at accessing and continuously improving the quality of patient care.
91. **Permits/permitting** is the process that a health facility must undergo to ensure it meets the standards of an oversight body or agency (often at arms-length from the government) that allow it to operate and to provide health services.
92. **Pharmacovigilance (system, centres, committees)** are activities for monitoring the safety of medicines and the detection, assessment, understanding and prevention of adverse effects or any other drug-related problem.
93. **Pharmacy and treatment committees** refers to a committee responsible for managing drug-related issues for the hospital(s) or other facilities represented. Typically, these committees focus on establishing and maintaining facility formularies, but may also be responsible for monitoring of adverse drug events in the hospital, development of clinical care guidelines, drug-use evaluation, establishment of therapeutic interchange policies, evaluation of medication-related patient safety issues, and management of product shortages *(13).*
94. **Plan-Do-Study-Act** is a cycle for continuous quality improvement and describes activities advocated for achieving processes of improvement: analyse the problem to be improved and devise a plan to correct the problem; carry out the plan; study the success of solving the problem; adopt the change piloted, abandon it if a failure or modify it and run through the cycle again *(5)*.
95. **Plan-do-study-act cycles (laboratories)** is a cycle for continuous quality improvement and describes activities advocated for achieving processes of improving the quality of laboratories: analyse the problem to be improved and devise a plan to correct the problem; carry out the plan; study the success of solving the problem; adopt the change piloted, abandon it if a failure or modify it and run through the cycle again *(5)*.
96. **Pre-admission patient data sharing** refers to the process of relaying information from an emergency medical vehicle to the emergency department that will intake the patient. This process allows for health professionals and hospital staff to be prepared for incoming patients and any care directives that need to be considered.
97. **Price controls** is the regulation of pharmaceutical pricing directly or indirectly through the mandates of social insurance schemes.
98. **Professional associations (bodies, councils, chambers)** represents the interests of health professionals and specialties by supporting national health policy development, engaging in negotiations on pay and working conditions of members, supporting continuous professional development, developing undergraduate and post-graduate education curricula and/or the development of clinical practice guidelines. This role is distinguished from health professional regulators, representing the interests of patients *(35)*.
99. **Professional certification** refers to the process by which an authorized body, either governmental or nongovernmental organization, evaluates and recognizes an individual health professional as meeting pre-determined requirements or criteria. It implies that the individual has received additional education and training and demonstrated competence in a specialty area beyond the minimum requirements set for certification *(13), (27), (28)*.
100. **Professional re-certification** refers to the process by which an authorized body, either governmental or nongovernmental organization, re-evaluates and recognizes an individual health professional as meeting pre-determined requirements or criteria after a set period of time (e.g. 5 or 10-year interval).
101. **Professional licensing** defines the process by which a governmental authority grants permission, usually following inspection against minimal statutory standards, to an individual practitioner or healthcare organization to operate or to engage in an occupation or profession. Licensure to individuals is usually granted after some form of examination or proof of education and maybe renewed periodically.
102. **Professional re-validation** is the process by which doctors have to regularly show that they are up to date and fit to practice medicine *(45).*
103. **Professional self-regulation** refers to a governance structure of health professionals whereby they are involved in determining the rules that govern their own profession and are accountable for their own behaviour with assistance and oversight provided by a regulatory body made up of members from the given profession.
104. **Public reporting on health professionals** refers to providing consumers, patients and other health system stakeholders with information about the performance of individual providers that allows them to judge their quality according to a given set of measures *(46).*
105. **Public reporting on performance by facility** refers to providing consumers, patients, providers and other health system stakeholders with information about the performance of facilities on a given set of measures *(46).*
106. **Quality improvement collaborative across facilities** a step-by-step approach to improve quality in a given area where there is a significant gap between best and current practice. The approach involves convening a group of interprofessional teams from across facilities to set measurable targets based on best practices and for them to test changes on a small scale within their facilities and to collaborate across facilities throughout out the process to share ideas, experiences and lessons learned.
107. **Quality improvement teams** refers to a group of individuals within a practice charged with carrying out improvement efforts. The team may report to management. Teams should meet regularly to review performance data, identify areas in need of improvement and carry out and monitor improvement efforts *(47, 48)*.
108. **Quality (service) report cards** are an assessment of the quality of services delivered and provide information on how well services respond to health needs according to a set of measures. Quality report cards can be published by regions (oblasts), insurers, consumer groups or other health organizations *(49)*.
109. **Regulation of market entry** are the range of conditions, tariffs and non-tariff measures (e.g., preference for domestic bidders; language requirements on packaging etc.) that may be put in place by governments to determine what organizations and what medicines are able to be sold (or enter the market) in any given jurisdiction.
110. **Report cards of health professionals** are a means to publicly compare performance measures of health professionals. Report cards of health professionals can support individuals in choosing among health professionals and motivate individuals to improve the quality of their services.
111. **Risk assessment** a process carried out prior to risky operations or conducting tests with reactive materials, during planning for the laboratory and before the purchasing of any new equipment, to safeguard those working in the laboratory from any harm as well as to protect the integrity of samples, equipment and premises.
112. **Root cause analysis** is an error analysis tool used to identify underlying problems that increase the likelihood of errors while avoiding the trap of focusing on mistakes by individuals *(50).*
113. **Sample registry** is a list of all patient samples previously and currently held by the laboratory.
114. **Self-assessment (self-audit)** the systematic process of self-collection of personal performance data, reflection on gaps between performance and standards and development and implementation of learning or quality improvement plans by individual health professionals *(51)*.
115. **Self-management** is a formalized strategy, protocol or standard for increasing the knowledge, skills and confidence to manage one’s own health, to care for a specific condition or to recover from an episode of ill health *(52),(14)*.
116. **Shared decision-making (strategy, protocol or standard)** are formalized to support patients, their families and carers, in collaboration with their health provider(s), to choose the next action(s) in their care path following an informed analysis of possible options, their values and preferences *(52),(14).*
117. **Simulation training (including standardized patients)** is a form of practice, training, monitoring or evaluation of capabilities involving the description or simulation of a clinical circumstance to which a described or simulated response is made *(53).*
118. **Standard operating procedures** are detailed instructions about how to undertake the testing or examination process. They provide step-by-step instructions for the laboratory staff to follow for each activity.
119. **Standard procurement processes** is a consistent approach used to evaluate the vendors from whom to purchase laboratory equipment. This approach may include defining criteria for supplies or materials to be purchased, considering the prices offered by different vendors, and any advantages and disadvantages to purchasing brand name compared to generic products *(9).*
120. **Standardized handover forms** are template forms used to transfer patients between professionals at shift changes or during consultations. They ensure that consistent information is collected and passed-on between professionals and allow for discussion of pertinent aspects of patient care.
121. **Standardized test request forms** are a template used to submit requests for patient lab testing, and typically contains patient details, requester details, sample details, the type of test required and any relevant clinical information.
122. **Structured clinical vignettes** are a method used measure and evaluate trainees’ knowledge and clinical reasoning. The vignettes are structured to provide the trainees with a description of a specific clinical situation followed by a series of questions prompting the trainee to explain how they would care for the patient given the information presented. Vignettes may be published in training texts or be provided as short videos as part of training materials.
123. **Structured medication review/reconciliation** is the process of reviewing medication regimens for individual’s with long-term conditions or taking multiple medications to identify opportunities for improvement and ensure accurate and complete medication information transfer at interfaces of care e.g. change in dosage, new medicines needed, etc. *(54)(55).*
124. **Supply chain management** encompasses the planning and management of all activities involved in sourcing and procurement. Importantly it also includes coordination and collaboration with channel partners *(56).*
125. **Task-shifting** involves the rational redistribution of tasks among health workforce teams, where appropriate, from highly qualified health workers to health workers with shorter training and fewer qualifications in order to make more efficient use of the available human resources for health *(57).*
126. **Team changes** are shifts to the orientation or composition of a multidisciplinary health team.
127. Training and education is a general term used for one-off efforts to inform providers or to change their behaviours, this is contrasted against more formalized efforts such as continuing medical education.
128. Unique patient identifier in health information systems refers to the use of information such as a person’s date of birth and part of their identification number (e.g. social security number) to create a unique code that is reported instead of a name. Unique patient identifiers can support privacy protection, individual control of information and improved access to patient information.

**Supplementary file 3: AMSTAR (quality) rating applied**

Assessing the methodological quality of systematic reviews (AMSTAR) is a commonly used tool to assess the quality of systematic reviews. AMSTAR ratings are based out of 11, where 11/11 is the highest quality. Scores between 8 and 11 out of 11 are considered to be of high- quality, whereas scores between 4 and 7 out of 11 are considered to be of medium quality and scores of 3 and under are considered of low-quality. The rating of a low-quality review does not mean it should be discounted, but that less confidence can be placed in the findings and the review’s limitations should be considered when drawing conclusions. For some systematic reviews, mostly, the denominator will be less than 11 (e.g. 10 or 9), as two of the 11 questions for scoring rely on being able to pool results. For this scoping review, these scores were used to help assign an evidence statement in the table below to each of the mechanisms and are reported in the quality and recency column in Supplementary file 4. The criteria for each of the evidence statements below were developed beforehand and consistently applied to the findings from systematic reviews included for each of the mechanisms.

**Table SF3.** Types of evidence statements and the level of evidence required to support the statement

| **Evidence statement** | **Level of evidence required** |
| --- | --- |
| Sufficient review-level evidence to either support or discount the effectiveness of the mechanism on quality | - Clear and consistent message coming from included high or medium-quality reviews - Reviews included methodologically robust studies |
| Tentative review-level evidence to either support or discount the effectiveness of the mechanism on quality | - A tentative statement from most of the included reviews - Consistent evidence from a small number of reviews containing a small number of studies (of varying quality) - Conflicting (or mixed) evidence from one or more reviews included, with the stronger evidence weighted towards one side |
| Insufficient review-level evidence to either support or discount the effectiveness of the mechanism on quality | - A statement of insufficient evidence from an included review - Conflicting (or mixed) evidence from one or more reviews included - No reviews on the mechanism, possibly due to a lack of robust primary studies available |

Source: Adapted from *(58)*

**Supplementary file 4: Summary of evidence on effectiveness of quality of care mechanisms**

| **Continuum** | | **Mechanism** | **Statement from review(s)** | **Quality and recency of evidence** | **Evidence statement** |
| --- | --- | --- | --- | --- | --- |
| Empowering people | Input-oriented | Citizens’ panels/juries | Sarrami-Foushani et al. did not identify any studies examining the effectiveness of citizen panels (or juries) on quality of care *(59)*.  Mitton et al. did not identify any studies examining the effectiveness of citizen panels (or juries) on quality of care *(60)*.  Street et al. did not examine the effectiveness of citizen panels on improving quality of care but identified a number of common elements across citizen juries/panels.(10) | One recent and two older medium-quality reviews. | Insufficient review-level evidence to either support or discount the effectiveness of the mechanism. |
|  |  | Consumer association (groups) | No reviews found | No reviews found. | Insufficient review-level evidence to either support or discount the effectiveness of the mechanism. |
|  |  | Consumer directed information | Kurtzman et al. did not find studies that examined the effectiveness of consumer information on quality of care however, the review found that consumers better respond and are better able to make informed choices when information provided in simple, presented in a positive manner, uses non-technical language, and makes the context of the information relevant to the consumer.(61) | One recent medium-quality review | Insufficient review-level evidence to either support or discount the effectiveness of the mechanism. |
|  |  | Health service ombudsman | Wager et al. found the existence of ombudsman can have an impact on patient-related outcomes but found not relationship with compliance to standards *(62)*. | One older medium-quality systematic review. | Insufficient review-level evidence to either support or discount the effectiveness of the mechanism. |
|  |  | Participation of community representatives in decision-making | Sarrami-Foroushami et al. found that the success of community engagement is dependent upon the approach taken and contextual factors. The review found a wide range of different approaches and methods for community engagement that limit conclusive findings.  Nilsen et al. found little evidence from five comparative studies of the effects of consumer involvement in health care decisions at the population level. (63)  Conkline et al, was unable to draw conclusions on the effectiveness of consumer involvement in the development of effective policy.(64) | One recent medium-quality, one older medium-quality, and one older high-quality review. | Insufficient review-level evidence to either support or discount the effectiveness of the mechanism. |
|  | Process-oriented | Consumer education | Ranji et al. found that consumer education alone on antibiotic use and medication safety did not improve quality of care, however may be effective when combined with additional interventions. Only two studies were included in the review examining consumer education alone, limiting conclusive findings.(65)  Nilsen et al. found that educational materials developed in partnership with consumers may improve knowledge about a given health issue.(63) | One older medium-quality and one older high-quality review. | Insufficient review-level evidence to either support or discount the effectiveness of the mechanism. |
|  |  | Consumer watchdog committee | No reviews found. | No reviews found. | Insufficient review-level evidence to either support or discount the effectiveness of the mechanism. |
| Engaging patients | Input-oriented | Patient association | No reviews found. | No reviews found. | Insufficient review-level evidence to either support or discount the effectiveness of the mechanism. |
|  |  | Patient bill of rights (patient charter) | No reviews found. | No reviews found. | Insufficient review-level evidence to either support or discount the effectiveness of the mechanism. |
|  | Process-oriented | Automated alerts and reminders for patients | Walsh et al. found patient reminders had little effect on either systolic or diastolic blood pressure among hypertensive patients.(66)  Shojania et al. found some reduction in diabetes patients’ HbA1c levels but relatively little effect on incremental reductions.(67)  Cabello et al. did not report on the effectiveness of individual quality improvement interventions, but found that interventions which focused on organizational structures and resources were more likely to be successful than patient education.(68)  Ovretveit found in one overview relatively little empirical evidence to support the use of patient reminders (mail and telephone) to reduce non-adherence. Similarly, small results were found for reminder packaging of medications, however when coupled with education, calendar packaging may improve adherence.  Scott found modest effects from the use of patient alerts and reminders, but found that these effects were limited to screening and vaccines in primary care.(69) | One recent high-quality, one recent, two older medium-quality, and one older low-quality reviews. | Tentative review-level evidence to discount the effectiveness of the mechanism when implemented on its own. |
|  |  | Patient complaint systems | No reviews found. | No reviews found. | Insufficient review-level evidence to either support or discount the effectiveness of the mechanism. |
|  |  | Patient pathways (care pathway, care map) | Rotter et al found that in 12 (of 16) studies the implementation of patient pathways shortened the length of hospital stays but had no significant effect on readmissions to hospitals or in-hospital complications.(70)  Scott found no change in mortality, dependency, discharge or length of stay from a review of 10 trials but another review found pathways increased the frequency of use of guideline-recommended treatments.(69) | One older high-quality review and one older low-quality reviews. | Insufficient review-level evidence to either support or discount the effectiveness of the mechanism on quality. |
|  |  | Patient decision-aids (supports) | Sarrami-Foroushani et al. found decision aids useful to the patient if they have more than one option, when treatment outcome is based on compliance or when patient’s need more knowledge about procedures or treatment *(59)*.  Ovretveit found positive results from the use of patient decision aids in one overview, including improved communication with health providers, however some variable effects were found for specific decision aids *(71)*.  Scott found in a review of 33 trials that decisions aids had no impact on patient satisfaction or health outcomes, but were found to reduce decisional conflict and lead to more realistic views on treatment effects (69). | One recent medium-quality, one older medium-quality, and one older low-quality review. | Tentative review level evidence to support the effectiveness of the mechanism. |
|  |  | Patient education | Ranji et al. found that patient education on its own had a small effect on improving the use of antibiotics, however may be more effective when coupled with other interventions such as audit and feedback and provider education.(65)  Colla et al. found evidence to support the use of direct patient education, reporting that while evidence is limited, there is some support that patient education can affect patient perceptions and change behavior around certain types of care.(72)  Shojonia et al. found patient education for diabetes were effective in improving process of care though had little impact on patient outcomes.(67)  McMurchy et al. found that patient education may help to increase patient knowledge and engagement in care but did not report on changes to patient outcomes.(46)  Faden et al. examined consumer education about pharmaceutical prices in LMIC and found a paucity of evidence but some findings to indicate that educated patients were more likely to adhere to medication regiments.(73) | One older high-quality, one recent medium-quality, two older medium-quality, and two lower-quality review. | Insufficient review-level evidence to either support or discount the effectiveness of the mechanism. |
|  |  | Peer support groups (peer-to-peer supports) | Patil et al. found peer support groups included in 17 studies resulted in a small but significant improvement in HbA1c levels among diabetic patients.(74)  Dale et al. found peer support calls in six studies were associated with increased uptake of screenings and may be associated with supporting changes in health behaviour but had no effect on health status, self-efficacy or mental health outcomes. Additional research is required to make a conclusion about the association, given methodological limitations in the included studies.(75) | One older and one recent high-quality review. | Tentative review-level evidence to support the effectiveness of the mechanism for select chronic conditions. |
|  |  | Self-management | Walsh et al. found that self-management among hypertensive patients had a statistically significant effect on improving diastolic blood pressure but not systolic. It is thought this might be a result of the historical focus on DBP in management of hypertension.(66)  Shojania et al found no significant change in HbA1c levels among diabetic patients as a result of implementing self-management as a quality improvement mechanism.(67)  Cabello et al. did not report on the effectiveness of individual quality improvement interventions, but found that interventions which focused on organizational structures and resources were more likely to be successful than patient education.(68)  Ryan et al. found evidence that self-management of anti-thrombotics is generally effective and some evidence that self-management may improve clinical outcomes in improves clinical outcomes related to thromboembolic events but insufficient evidence related to self-management of major haemorages.(76) | One overview of systematic reviews, two older medium-quality, and one recent high-quality review. | Tentative review-level evidence to support self-management to improve the quality of care for select conditions. |
|  |  | Shared decision-making | Ovretveit et al. found that while shared-decision making resulted in improvements in care processes, training physicians to use shared decision-making was unlikely to result in lower costs or improved health outcomes.(71) | One older medium-quality. | Insufficient review-level evidence to either support or discount the effectiveness of the mechanism. |
|  | Output-oriented | Patient feedback system | Cheraghi-Sohi and Bower found mixed-effects from limited evidence (two studies) about the effects of patient feedback on improving the interpersonal skills of primary care physicians.(77) | One older medium-quality review. | Insufficient review-level evidence to either support or discount the effectiveness of the mechanism. |
|  |  | Patient reported experience surveys | No reviews found. | No reviews found. | Insufficient review-level evidence to either support or discount the effectiveness of the mechanism on improving quality. |
|  |  | Patient satisfaction surveys | Ridd et al. found that across 11 studies, satisfaction surveys did not fully capture the essence of the therapeutic relationship or adequately measure many of the aspects that affect patients quality of care.(78) | One older medium-quality review. | Insufficient review-level evidence to either support or discount the effectiveness of the mechanism on improving quality. |
|  | Outcome-oriented | Patient reported outcome surveys | Kotronoulas et al. found tentative evidence from 26 studies that the use of patient reported outcomes may improve quality of cancer care, with the greatest amount of evidence supporting improves in patient satisfaction from communication with physicians.(79)  de Almeida et al. found significant variation among patient reported outcome surveys and their reliability and validity to measure the multiple dimensions of patient outcomes and experience of care. The review found that overall they were found to result in small to moderate improvements in patient safety and quality of care, however the reviews noted that more evidence is required and greater standardization among surveys used.(80) | One recent high-quality and one recent medium-quality reviews. | Tentative review-level evidence to support the effectiveness of the mechanism. |
| Improving clinical practice | Input-oriented | Clinical practice standards | Hughes et al. found in one systematic review that the simplification and standardization of clinical processes (e.g., strategies that reduce the reliance on individualize decision-making) improved patient outcomes, efficiency and effectiveness.(81)  Sutherland et al. found four studies which reported an association between standards and improvements in quality.(82) | Two older overviews of systematic reviews. | Tentative review-level evidence to support the effectiveness of the mechanism on quality. |
|  |  | Clinical protocols and guidelines | Manias et al. found protocols and guidelines reduced the number of incompatible medication pairs and of ambiguous errors following the implementation of standardized order sheets and prescription guidelines.(83)  Willey et al. found in one study that the implementation of guidelines, when delivered alongside other quality of care mechanisms (e.g., training, supervision, progress surveys and feedback) improved quality of care.(84)  Ament et al. found that mixed results regarding the sustainability of guidelines once implemented. The review found they were sustained in less than half of the studies included in the review.(85)  Scott found clinical practice guidelines improved processes of care in 46 (of 59) studies and improved patient outcomes in 9 (of 11). The review found that clinical practice guidelines were most effective when they are adapted to local needs, disseminated by an active educational intervention, presented in easy to use way, and implemented using patient specific reminders.(69)  White et al. found the implementation of guidelines by quality improvement teams in 9 of 16 studies to improve quality, but only three of these studies reported sustained changes over the long-term.(86)  Dayal et al. found no evidence to support clinical guideline dissemination as a stand-alone intervention in low-income ambulatory care settings. However, when combined with other mechanisms such as educational outreach, reminders and audit and feedback can result in small improvements to practice.(87)  Lugtenberg et al. found the implementation of evidence-based clinical practice guidelines improved both the process and structure of care and resulted in small improvements in patient outcomes.(88) | One overview of systematic reviews, two older high-quality review, one recent medium-quality review, two older medium-quality reviews, and one older low-quality review. | Sufficient review-level evidence to support the effectiveness of the mechanism on quality when implemented alongside other mechanisms. |
|  |  | Disease registries | No reviews found. | No reviews found. | Insufficient review-level evidence to either support or discount the effectiveness of the mechanism on quality. |
|  | Process-oriented | Clinical checklists | Lyons and Popejoy found the use of surgical checklists improved teamwork and communication, reduced morbidity and mortality and improved compliance with safety measures, however these findings should not be generalized to other clinical settings.(89) | One recent low-quality review. | Insufficient review-level evidence to either support or discount the effectiveness of the mechanism on quality. |
|  |  | Clinical decision support systems (incl. alerts and reminders) | Manias et al. found mixed results from clinical decision support systems whereby four studies found a reduction in medication errors while the remaining two studies demonstrated no change.(83)  Camire et al. found positive results from one study on adherence to suggestions provided by clinical decision supports systems, reductions in prescriptions that patients were allergic to, and a reduction in antibiotic-susceptibility mismatches.(90)  Willey et al. found positive results from one study that reminders to facility health workers for the management of non-severe malaria improve the quality of care.(84)  Marcum et al. found mixed results from two studies about the effects of clinical decision support systems on adverse events. The review found that while they did not reduce the rate of adverse effects they may improve aspects of prescribing such as administration frequency and raise clinician’s awareness to what medications should be avoided.(91)  Sketris et al. found in one systematic review reminders and decision supports to have a moderate effect on improving physician prescribing practices according to guidelines and improvements in patient outcomes. The review emphasized that the effectiveness of reminders and decisions supports likely depends on a combination of the physicians’ characteristics and clinical environment.(92)  Robertson et al. found among 21 studies that clinical decision support systems were effective at improving one dimension of care (e.g., prescribing, clinical or patient). Support systems that were implemented to ensure patient safety were more effective than those focused on the quality use of medicines.(93)  Scott found significant evidence across systematic reviews to support the implementation of clinical decision support systems, finding significant increases in guideline-adherent care and improvements in practice. Clinical decision support systems were most effect when systems used reminder formats, targeted drug prescribing, automatically prompted physicians, and used software acceptable to physicians.(69)  Colla et al. found that decision supports integrated in electronic health records have significant potential to reduce the use of low-value care.(72)  Lu et al. found that the use of faxed reminders had no discernable effect on the proportion of non-adherent patients, however computerized reminders have demonstrated some effectiveness in improving rates of laboratory drug monitoring.(94)  McMurchy et al found that the use of reminders and clinical decision-support systems in primary care has improved operational efficiency and quality of care, including improved screening, decreased prescribing errors.(46) | One older high-quality, one recent medium-quality, six older medium-quality, and two older low-quality reviews. | Sufficient evidence to support the effectiveness of the mechanism on quality. |
|  |  | Computerized diagnostics | McDonald et al. found 32 studies that examined technology-based systems and reported beneficial effects in reducing diagnostic error following computer based diagnosis and decision-making interventions.(95) | One recent medium-quality review. | Tentative review-level evidence to support the effectiveness of the mechanism on quality. |
|  |  | Delayed prescribing | Ranji et al. found that among five studies there was a marked reduction in the use of antibiotics following the implementation of delayed prescribing (which in two studies was combined with patient education). However, the review points to the possibility that delayed prescribing strategies appear more effective because of extremely high rates of antibiotic use in the control group.(65) | One older medium-quality review. | Insufficient review-level evidence to either support or discount the effectiveness of the mechanism on quality. |
|  |  | Structured clinical vignettes | No reviews found. | No reviews found. | Insufficient review-level evidence to either support or discount the effectiveness of the mechanism on quality. |
|  |  | Facilitated relay of clinical data to providers | Ricci-Cabello et al. found that when combined with other health system interventions including case management, team changes and electronic patient registry were found to be highly effective in changing processes of care, diabetes self-management, and clinical outcomes.(68)  Shojania et al. found facilitated relay to be more effective on reduce HbA1c l compared to controls among patients with diabetes.(67) | One older high-quality and one older medium-quality review. | Tentative review-level evidence to support the effectiveness of the mechanism. |
|  |  | Standardized medication reports | Laugaland et al. found positive results from two studies that the use of standardized or structured medication reviews reduced the number of medication errors and adverse clinical consequences among the elderly.(96) | One older low-quality review. | Tentative review-level evidence to support the effectiveness of the mechanism on quality. |
|  |  | Structured medication review/reconciliation | Manias et al. found inconclusive results from one study on using a discharge survey as part of a medication reconciliation.(83)  Camire et al. found positive results on a reduction in medication errors from one study on medication reconciliation.(90)  Chistensen et al found no evidence from 10 studies that medication reviews reduced mortality or hospital readmission, however found some evidence that their use may the number of emergency contacts.(97)  Hammad et al. found some positive results from 13 studies examining pharmacy-led medication reviews which in some cases led to a reduction in hospital readmissions and emergency department visits, however had no impact on mortality and uncertain effects on costs. Authors point to more research being needed before drawing a conclusion about the effectiveness of structured medication reviews.(98) | Two recent high-quality review and two older medium-quality review. | Tentative review-level evidence to support the effectiveness of the mechanism on quality. |
|  | Output oriented | Audit and feedback | Chaudhuri et al. found audit and feedback for clinicians was strongly associated with reductions in inappropriate testing. However, these findings were not sustained over the long-term (e.g., 24 months).(99)  O’Beirn et al. found the implementation of audit and feedback mechanisms in primary care increase the use of preventative services (e.g., mammography, tobacco cessation, fecal occult blood screening).(100)  Willey et al. found one systematic review (118 studies) that reported they found no evidence to suggest the use of audit and feedback as a mandatory intervention to improve health worker behaviour.(84)  Sketris et al. found mixed effects on the use of audit and feedback to improve professional practice, with the largest effects found when baseline levels of adherence to standards and guidelines was low.(92)  Scott found a significant amount of evidence supporting the use of audit and feedback mechanisms, which averaged a 5% increase in reporting outcomes for dichotomous measures and 16% for studies with continuous variables in a review of 118 studies. In line with other evidence, audit and feedback is most effective when baseline adherence to recommended care is low.(69)  Dayal et al. found audit and feedback had a small to moderate impact on improving the quality of care. Authors noted that it is difficult to implement in low and middle-income countries given the need for good routine data collection.(87)  Colla et al. found that the use of feedback with professionals on their compliance to guidelines has been shown to significantly decrease inappropriate ordering of imaging and medicines.(72)  Lu et al. found in four studies that audit and feedback could achieve small to moderate improvements in physicians practice, including changes to medication prescriptions.(94)  Sykes et al. found a significant improvement in 13 studies  in the dementia care provided by health professionals. However, all of the studies included had significant methodological limitations. (101) | One overview of systematic reviews, two recent high-quality, one older high-quality, one recent medium-quality, three older medium-quality, and one older low-quality review. | Sufficient review-level evidence to support the effectiveness of the mechanism on quality. |
|  |  | Critical Incident Report/Adverse event reporting | Brunsveld-reinders et al. found significant variation in the types of incident reporting systems being used, but that they may be effective in ICUs when a multidisciplinary approach to implementation is used and/or the presence of regular feedback meetings and report sessions.(102)  Hughes et al. found mixed evidence about the use of error reporting suggesting that while it may be effective when implemented under the right conditions, providers often hesitated to report errors and as a result significantly reduced the effectiveness.(81) | One overview of systematic reviews and one older medium-quality review. | Insufficient review-level evidence to either support or discount the effectiveness of the mechanism on quality. |
|  |  | Patient safety reporting | No reviews found. | No reviews found. | Insufficient review-level evidence to either support or discount the effectiveness of the mechanism on quality. |
|  | Outcome-oriented | Morbidity and mortality reviews | Bal et al. found some evidence to support the use of morbidity and mortality reviews to improve providers knowledge and quality of care, however the review found significant variation between the studies and methods included and suggest the development of a consistent methods for evaluating the use of morbidity and mortality reviews.(103) | One older low-quality review | Insufficient review-level evidence to either support or discount the effectiveness of the mechanism on quality. |
|  |  | Never ‘event’ reporting | No reviews found. | No reviews found. | Insufficient review-level evidence to either support or discount the effectiveness of the mechanism on quality. |
| Ensuring responsive emergency medicine (ambulance) | Input-oriented | Dispatch protocols | McQueen et al. found a non-significant reduction in the length of time to reach incident scenes of four minutes when dispatch protocols were used and no effect on mortality, severity of injury or proportion of patients admitted to intensive care. However, this finding comes from one study.(104) | One recent medium-quality review | Insufficient review-level evidence to either support or discount the effectiveness of the mechanism on quality |
|  | Process-oriented | Care pathways including transfer pathways | Fisher et al. notes the increase in use of ATMIST handover procedures in response to extensive literature around the challenges in consistent handovers, however the review found that there is a need for studies to investigate the extent to which these standardized handover protocols reduce accidents(104) | One older high-quality review | Tentative review-level evidence to support the effectiveness of the mechanism on quality |
|  |  | Computerized decision support systems (incl. triage) | Fisher et al. found little published peer-reviewed evidence on the effectiveness of computerized decision support systems on improving quality, however suggested this evidence is likely in progress given the recent adoption of these systems.(104) | One older high-quality review | Insufficient review-level evidence to support the effectiveness of the mechanism on quality |
|  |  | Dosing/code card | Bigham et al. found dosing cards improved the accuracy of medication doses and estimates of endotracheal tube sizes, significantly reducing error rates.(105) | One older medium-quality review | Tentative review-level evidence to support the effectiveness of the mechanism on quality |
|  |  | Equipment checklist | Fisher et al. found that the use of checklists has shown some promise in avoiding patient safety incidents resulting from equipment failure (104) | One older high-quality review | Sufficient review level evidence to support the effectiveness of the mechanism on quality |
|  |  | Pre-admission patient data sharing | Fisher et al. found the sharing of pre-admissions data (particularly ECG results) to determine where patient’s should be directed reduced time to reperfusuion, however the review notes that depending on the training of paramedics this may not be necessary.(104)  Synnot et al. examined the use of prehospital notification for major trauma patients requiring emergency hospital transport and found limited evidence, with only two studies included in the review. One of which found a significant decrease in mortality following notification, while the other found no overall effect.(106) | One recent and one older high-quality reviews | Tentative review-level evidence to support the effectiveness of the mechanism on quality |
|  |  | Standardized handover forms | Bost et al include three studies in the review that recommend the use of standardized handover forms as a method to improve patient safety in handovers between ambulance services and emergency rooms. The review however notes that the standardized handover model needs to be flexible to fit within the context of a patient, local environment and organizational culture.(107) | One older medium-quality | Tentative review level evidence to support the effectiveness of the mechanism on quality |
|  | Output-oriented | Critical incident reporting | Fisher et al. found a lack of research that assessed whether critical incident reporting was taking place in ambulance services and that more research is needed to directly measure aspects of safety culture.(104)  Bigham et al. examined self-reporting and found that paramedics were more likely than nurses to self-report an error but less likely than physicians. Paramedics noted that for an environment to encourage self-reporting cultures had to be non-punitive.(105) | One older high-quality review and one older medium-quality review | Insufficient review-level evidence to either support or discount the mechanism on quality |
| Enhancing laboratory services | Input-oriented | Accreditation | No reviews found | No reviews found | Insufficient review-level evidence to either support or discount the effectiveness of the mechanism on quality. |
|  |  | Certification | No reviews found | No reviews found | Insufficient review-level evidence to either support or discount the effectiveness of the mechanism on quality. |
|  |  | Computerized inventory management | No reviews found | No reviews found | Insufficient review-level evidence to either support or discount the effectiveness of the mechanism on quality. |
|  |  | International laboratory standards | No reviews found. | No reviews found. | Insufficient review-level evidence to either support or discount the effectiveness of the mechanism on quality. |
|  |  | Internationally recognized labels | No reviews found | No reviews found | Insufficient review-level evidence to either support or discount the effectiveness of the mechanism on quality. |
|  |  | Licensure | No reviews found. | No reviews found. | Insufficient review-level evidence to either support or discount the effectiveness of the mechanism on quality. |
|  |  | Patient identifiers and sample identifiers | Snyder et al. found that individual bar-codes for patient specimens was effective for reducing patient specimen and laboratory testing identification errors in diverse hospital settings (108) | One older high-quality review | Sufficient review level evidence to support the effectiveness of the mechanism on quality when implemented in high-income countries |
|  |  | Sample registry | No reviews found | No reviews found | Insufficient review-level evidence to either support or discount the effectiveness of the mechanism on quality. |
|  |  | Standard purchasing or procurement process | No reviews found | No reviews found | Insufficient review-level evidence to either support or discount the effectiveness of the mechanism on quality. |
|  |  | Standardized test request forms | No reviews found | No reviews found | Insufficient review-level evidence to either support or discount the effectiveness of the mechanism on quality. |
|  | Process-oriented | Circulation pathways | No reviews found | No reviews found | Insufficient review-level evidence to either support or discount the effectiveness of the mechanism on quality. |
|  |  | Computerized order entry | Manias et al, found mixed results for computerized order entry as it was found in three of five studies to increase the total number of reported medication errors following implementation but reduced the number of errors that did harm to patients.(83)  Camire et al. found from three studies and that computerized order entry reduces the number of medication errors among elderly patients in the ICU.(90)  Lu et al. found in one systematic review that computerized order entry with clinical decision support improved prescribing and reduced medication error rates. (94)  Nuckols et al. found in a meta-analysis of 13 studies that computerized order entry was associated with a 50% reduction in preventable adverse drug events.(109) | One recent high-quality review and three older medium-quality reviews | Sufficient review-level evidence to support the effectiveness of the mechanism on quality |
|  |  | Equipment inventory list | No reviews found. | No reviews found. | Insufficient review-level evidence to either support or discount the effectiveness of the mechanism on quality. |
|  |  | Equipment validation and function checks | No reviews found. | No reviews found. | Insufficient review-level evidence to either support or discount the effectiveness of the mechanism on quality. |
|  |  | Risk assessment | No reviews found | No reviews found | Insufficient review-level evidence to either support or discount the effectiveness of the mechanism on quality. |
|  |  | Standard operating procedures | No reviews found | No reviews found | Insufficient review-level evidence to either support or discount the effectiveness of the mechanism on quality. |
|  | Output-oriented | External quality assessment | No reviews found | No reviews found | Insufficient review-level evidence to either support or discount the effectiveness of the mechanism on quality. |
|  |  | Laboratory quality indicators | No reviews found | No reviews found | Insufficient review-level evidence to either support or discount the effectiveness of the mechanism on quality. |
|  |  | Laboratory safety audits | No reviews found | No reviews found | Insufficient review-level evidence to either support or discount the effectiveness of the mechanism on quality. |
|  |  | Plan-do-study-act cycles | No reviews found | No reviews found | Insufficient review-level evidence to either support or discount the effectiveness of the mechanism on quality. |
| Managing services | Input-oriented | Facility performance agreements | McMurchy et al found that region and regional-service performance frameworks and agreements that are accompanied by evidence-based targets can support quality improvement, however the overview notes that quality of care and patient experience indicators have typically been missing from these agreements.(46) | One older low-quality review. | Tentative review-level evidence to support the effectiveness of the mechanism on quality. |
|  | Process-oriented | Failure Modes and Effects Analysis | Hughes et al. found that while health failure modes and effects analyses were effective as a proactive analysis in hospitals, they were found to be time consuming to undertake.(81) | One overview of systematic reviews. | Tentative review-level evidence to support the effectiveness of the mechanism on quality. |
|  |  | Quality improvement collaborative across facilities | Schouten et al. found a positive association between quality improvement collaboratives and improvements in process of care and care outcomes, however methodological limitations in the available literature limited conclusions about causation.(110)  McMurchy et al found that quality improvement collaboratives targeting diabetes and other chronic conditions have generally resulted in improved health outcomes, increased preventative procedures and reduced hospitalization.(46)  Well et al. found that quality improvement collaboratives resulted in significant improvements in targeted clinical processes and patient outcomes across settings, with eight o 39 studies reporting long-term persistence of results after the end of the collaborative (111) | One recent high-quality, one older medium-quality and one older low-quality review. | Sufficient review level evidence to support the effectiveness of the mechanism on quality. |
|  | Output-oriented | Benchmarking | McMurchy et al. found that benchmarking generally supported high-quality primary care however no results of effectiveness were reported. | One older low-quality review. | Insufficient review-level evidence to either support or discount the effectiveness of the mechanism on quality. |
|  |  | External benchmarking | Phillips et al. found national benchmarking may increase accessibility of services and capabilities of providers. The review found that there was no effect on responsiveness.(112) | One older low-quality review | Tentative review-level evidence to support the effectiveness of the mechanism on quality. |
|  |  | Internal benchmarking | No reviews found | No reviews found | Insufficient review-level evidence to either support or discount the effectiveness of the mechanism on quality. |
|  |  | Facility performance indicators | de Vos et al. included six studies on facility performance indicators which found mixed results, with four studies reporting no effect on quality, one was partially effective and in one study they were found to be effective in improving quality. The review found that the implementation of quality indicators in hospitals is most effective if feedback reports are given in combination with educational implementation strategies. No reviews found. (113) | One older low-quality review | Insufficient review-level evidence to either support or discount the effectiveness of the mechanism on quality, however may be effective when implemented in parallel with other quality mechanisms. . |
|  |  | Plan-Do-Study-Act | Nicolay et al. found some positive results for the use of plan-do-check-act methodologies including a reduced delay in the start of surgeries. Relatively few studies were found examining this mechanisms and those that were did not report statistical significance of outcomes. In addition, the methods used to conduct the studies have a high-risk of bias.(114)  Hughes et al found plan-do-study-act to be most effective when implemented as a series of rapid cycles and when implemented within a complex system.(81) | One overview of systematic reviews and one older medium-quality review. | Tentative-review level evidence to support the effectiveness of an intervention. |
|  |  | Quality improvement teams (quality circles) | Conry et al found quality intervention teams improved quality of care and outcomes for patients using multi-component interventions. However, these findings only applied to specific illnesses or conditions (e.g., pneumonia, diabetes, smoking cessation).(8)  O’Beirn et al. found mixed results from three studies on outcomes of care from quality improvement teams implementing disease specific multi-component interventions.(100)  White et al. found that across 45 studies while quality improvement teams had some positive results, methodological limitations in the studies included limited conclusions about the effectiveness of the mechanism.(86) | One older high-quality and two older medium-quality reviews, | Tentative review-level evidence to support the effectiveness of the mechanism on quality for targeted diseases. |
|  |  | Quality service report cards | No reviews found. | No reviews found. | Insufficient review-level evidence to either support or discount the effectiveness of the mechanism on quality. |
|  |  | Root Cause Analysis | Kurji et al. found some evidence to support the use of root-cause analyses in reducing adverse events, increasing patient safety and quality of care. It should be noted that no details were provided about the methods used in these studies and therefore the findings should be interpreted with caution.(115) | One recent low-quality systematic review. | Insufficient review-level evidence to either support or discount the effectiveness of the mechanism on quality. |
| Ensuring a competent health workforce | Input-oriented | Accreditation of certifying bodies | No reviews found. | No reviews found. | Insufficient review-level evidence to either support or discount the effectiveness of the mechanism on improving quality. |
|  |  | Accreditation of training schools | Greenfield et al. found a small but significant link between professional development and accredited training programs in four studies. The reviews found that professionals who attended accredited programs were more likely to pass exams and engage in ongoing professional education. However, the review found no significant effect on professional performance.(116) | One older low-quality review. | Tentative review-level evidence to discount the effectiveness of the mechanism on improving quality when implemented on its own. |
|  |  | Co-regulation | No reviews found. | No reviews found. | Insufficient review-level evidence to either support or discount the effectiveness of the mechanism on improving quality. |
|  |  | Educational outreach | Marcum et al. found mixed results for the use of educational outreach in the home and community care sector, with on study reporting no significant differences while two others reporting improvements in prescription, however in one of the three studies educational outreach was included as part of a multi-intervention approach.(91)  Sketris et al. found that educational outreach and other types of academic detailing were generally found to be effective but that these interventions are not often cost-effective and should be assessed within the context and environment of the intervention.(92) | Two older medium-quality reviews | Tentative review-level evidence to support the effectiveness of the mechanism on improving quality when implemented on its own. |
|  |  | Health professional registry | No reviews found. | No reviews found. | Insufficient review-level evidence to either support or discount the effectiveness of the mechanism on improving quality. |
|  |  | List of professional competencies | No reviews found. | No reviews found. | Insufficient review-level evidence to either support or discount the effectiveness of the mechanism on improving quality. |
|  |  | Professional associations | No reviews found. | No reviews found. | Insufficient review-level evidence to either support or discount the effectiveness of the mechanism on improving quality. |
|  |  | Professional licensing | No reviews found. | No reviews found. | Insufficient review-level evidence to either support or discount the effectiveness of the mechanism on improving quality. |
|  |  | Professional self-regulation | Dieleman et al. found one study which saw an improvement in the availability of essential dispensing materials following regulation of pharmacies.(117) | One older medium-quality review | Insufficient review-level evidence to either support or discount the effectiveness of the mechanism on improving quality. |
|  |  | Simulation based training (including standardized patients) | Manias et al. found simulation-based training for medication prescribers decreased medication administration error rates in the short term (e.g., 6-12 weeks).(83)  Murdoch et al. found the use of: high-fidelity human patient simulators; role play; and role play with a standardized patient following lecuture were all effective training tools and demonstrated significant results for improving training. The involvement of multiple professionals in these activities found improvements in achieving interprofessional competencies.(118)  Dilaveri et al. found in 12 studies that compared technological-simulations with control groups, simulation-based training for breast and pelvic exams significantly improved providers clinical skills. In particular, the review found providing feedback in simulations during breast exams and feedback and a standardized patient for pelvic exams significantly improved the effectiveness of the intervention.(119) | One recent high-quality review and two older medium-quality review. | Sufficient review-level evidence to support the effectiveness of the mechanism on quality. |
|  |  | Training and education | Ranji et al. found that while no intervention was superior, active education strategies were more effective than passive in improving the quality of antibiotic prescribing.(65)  Laugaland et al. found that transitional skills for the transfer of patients improved following integration into health professionals curriculum.  Conry et al. found that technical interventions which focused on improving care for patients with heart disease or pneumonia were more effective than interpersonal interventions. Education and training in patient care was included as a technical interventions however, specific findings on its effectiveness were not separated out.(8)  Lu et al. found that passive education was not effective at improving the quality and efficiency of medication use. The review found that active strategies such as academic detailing were effective and this is captured under the row on educational outreach.(94)  Manias et al. found that didactic education did not lead to improvements in medication error rates, however appeared to improve patient safety when combined with feedback.(83)  Rowe et al. found that training and education alone had moderate effects on improving quality of care among health professionals in low and middle-income countries | One recent high-quality, four older medium-quality and one older low-quality review | Sufficient review-level evidence to support the effectiveness of the mechanism on improving quality in the short-term and when implemented using a participatory approach. |
|  | Process-oriented | Clinical observation | Gordon et al. found that generally studies examining the use of observation as a teaching methods was effective in changing their attitudes about medical harm and patient safety, however the studies included in the review were provided with training prior to observation limiting the extent of conclusions about its impact alone.(120)  Craig found that direct observation of clinical practice in emergency medicine was well received by professionals and in two studies reportedly led to positive behavior changes, however the review noted the relatively high cost of the intervention.(121) | One older high-quality review and one older medium-quality review | Tentative review-level evidence to support the effectiveness of the mechanism on improving quality when implemented on its own. |
|  |  | Clinical supervision | Dieleman et al. found a significant increase in adherence to protocols and standard treatment guidelines following six to eight months of clinical supervision.(122)  Willey et al. found two studies examining supervision, one of which reported that supervision following training had no significant effect on intention-to treat but increased the number of observed consultations, while the other reported small benefits to knowledge and practice but insufficient evidence to support a particular form of supervision.(84)  Craig et al. found in four studies (of varied quality) clinical observation in the emergency department to be beneficial in terms of knowledge, skills and attitudes of training clinicians and a reduction in immediate complications, however no differences in patient outcomes were observed at 28 days. The review noted that despite improvements in patient care, the overall efficiency of the emergency department decreased as a result of the involvement of senior staff in observation.(121)  Dayal et al. found mixed effects on the use of managerial supervision as a mechanism to improve quality. The overview reported no or uncertain impacts in low- and middle-income countries, however noted that one included study reported positive outcomes on quality of care.(87)  Snowdon et al. found that clinical supervision was associated with effectiveness of care, with the review finding significant improvements in process of care, including compliance with processes that that enhanced patient health outcomes.(17)  Sharma et al. identified supervision as being essential to overcoming bottlenecks in labour and delivery services, noting that it is a critical element of multicomponent interventions, however did not report findings of effectiveness on improving quality of care.(123) | One overview of systematic reviews, one recent high-quality, older high-quality, one older medium-quality, one recent low-quality and two older low-quality review. | Sufficient review-level evidence to support the effectiveness of this mechanism on improve some aspects of quality of care in the short term |
|  |  | Continuous medical education | Dieleman et al. found that continuing medical education could improve knowledge, skills and performance of certain tasks in the short-term but had variable results in the medium or long-term dependent on the education’s design. The review found effectiveness of CME was improved when it included a participatory approach, locally applicable material and practise during or after the training.(122)  Scott found some moderate effects in changes to professional practice following continuous medical education, however this was only found to be effective when implemented in an interactive manner (e.g., small group, case based, interactive workshops).(69)  Cervero et al. found that continuous medial education improved physician performance and patient health outcomes in eight systematic reviews. However, the overview noted that continuous medical education had a greater effect on physician performance than on patient health outcomes. Similar to other literature, the overview noted it is most effective when interactive and makes use of many methods.(124) | One recent overview of systematic reviews and two older low-quality reviews. | Sufficient review-level evidence to support the effectiveness of the mechanism on improving quality in the short-term and when implemented using a participatory approach. |
|  |  | Objective Structured Clinical Examinations | Setyonugroho et al. generally found support from 25 studies about the validity and reliability of Objective Structured Clinical Examinations for testing clinical skills. While the OSCE was found to be widely used for assessing communication skills as well, the effectiveness of its use in improving or signalling the need for improvements in quality of care was not examined. (125) | One recent medium-quality review. | Insufficient review-level evidence to either support or discount the effectiveness of the mechanism on improving quality. |
|  |  | Peer-review teams/committees | No reviews found. | No reviews found. | Insufficient review-level evidence to either support or discount the effectiveness of the mechanism on quality. |
|  |  | Professional re-certification | Sutherland et al. found one systematic review and four studies that reported positive associations between certification of specialty physicians and higher quality care.(82) | One older overview of systematic reviews. | Tentative review-level evidence to support the effectiveness of the mechanism on improving quality. |
|  |  | Task shifting | Sharma et al. identified task shifting as a potential solution to health workforce bottlenecks in labour and delivery services in low and middle income countries, however did not report on the effectiveness.(123) | One recent medium-quality review | Tentative review-level evidence to support the effectiveness of the mechanism on improving quality. |
|  |  | Team changes | Ricci-Cabello et al. did not report on the effectiveness of team changes or interdisciplinary teams despite including them as a quality assurance mechanism.(68)  Shojania et al. found changes to the structure of the team providing care including by creating interdisciplinary teams led to improved patient outcomes with respect to diabetes care and HbA(1c) levels.(67)  Walsh et al. found that interventions that included team change were effective in improving the patient outcomes for hypertension care.(66)  Sharma et al. identified changes to the delivery team as being a potential solution to labour and delivery bottlenecks in low and middle income countries, however the review did not report on findings of effectiveness.(123) | Older high-quality, two older medium-quality, and one recent low-quality- review. | Sufficient review-level evidence to support the effectiveness of the mechanism on quality. |
|  | Output-oriented | Multi-source feedback assessment | Khalifa et al. found multi-source feedback to be a feasible, reliable and valid tool to measure some technical and non-technical competencies (e.g., leadership, communication, systems-based practice) in surgical practice.(40) | One recent low-quality review. | Tentative review-level evidence to support the effectiveness of the mechanism in surgical practice. |
|  |  | Public reporting on health professionals | McMurchy et al. found a link between public reporting and quality improvement but stressed that public reporting works best when it is aligned with existing performance agreements.(46)  Fung et al. found inconsistent evidence of effectiveness on public reporting on patient care performance.(126)  Totten et al. found that while there is little evidence to suggest that patients make changes based on public reporting, moderate evidence was found that providers engage in activities to improve quality when their performance is made public.(127)  Herrera et al. found that disclosing performance information ot patients and to the public likely encourages hospitals to implement quality improvement activities and likely leads individuals to select providers that have better quality ratings.(128)  Parker et al. found that evidence on the effects of public reporting of the quality of stroke care was extremely limited and could not draw definitive conclusions.(129)  Behrendt and Groene found some evidence to support the use of public reporting among surgeons to improve quality, however the review warned about the negative effect on access to surgery for high-risk patients and non-whites that may occur.(130) | One recent high-quality, one older high-quality, two older medium-quality, one recent medium-quality, and one older low-quality review | Tentative review-level evidence to support the effectiveness of the mechanism on quality |
|  |  | Report cards of health professionals | Colla et al. found there has been too little research on provider report cards to determine their effectiveness, but noted that for them to be effective at influencing patient choice they need to be easily understood and present key information to patients.(72)  Dayal et al. found that provider report cards are emerging as an effective strategy to inform patients’ decisions, but emphasized the need for clearly presented information as a requisite for these strategies to be successful.(87) | One recent overview of systematic reviews and one recent medium-quality review. | Insufficient review-level evidence to either support or discount the effectiveness of the mechanism on quality. |
|  |  | Self-assessment (self-audit) | Gagliardi et al. found six studies (of varying quality) reporting improvements in compliance with guidelines and improved patient outcomes as a result of regular self-audits. The authors note that this is largely reliant of self-reported information.(51) | One older low-quality review. | Insufficient review-level evidence to either support or discount the effectiveness of the mechanism on quality. |
| Promoting the responsible use of pharmaceuticals and medical products | Input-oriented | Barcoding of pharmaceuticals | Young et al. found mixed effectiveness of implementing barcoding to reduce medication administration errors with three studies strongly supporting its use and one study finding an increase in medication errors. The authors point out a paucity in the literature on barcoding noting that no randomized controlled trials have been developed despite the technology having been in use for two decades.(131) | One older low-quality review. | Insufficient review-level evidence to either support or discount the effectiveness of the mechanism on quality. |
|  |  | Bulk purchasing | Faden et al. found relatively little evidence on bulk purchasing in low and middle income countries and as result was unable to make definitive conclusions.(73) | One older low-quality review | Insufficient review-level evidence to either support or discount the effectiveness of the mechanism on quality. |
|  |  | Essential Medicines List | No reviews found | No reviews found | Insufficient review-level evidence to either support or discount the effectiveness of the mechanism on quality. |
|  |  | Mandatory inspection of new pharmaceuticals | No reviews found | No reviews found | Insufficient review-level evidence to either support or discount the effectiveness of the mechanism on quality. |
|  |  | Medical formulary | Lu et al. found that changes to an established formulary and found in four studies that physician prescription patterns follow closely changes to the formulary. In particular, prescriptions for an entire category of drug fell in two studies when an over-the-counter alternative was made available.(94)  Faden et al. found the implementation of formularies or medicines reimbursement lists may decrease medicines expenditures and improve the utilization of cost-effective medicines in low and middle income countries, however more research is required before conclusions may be made.(73) | One older medium-quality and one older low-quality review. | Tentative review-level evidence to support the effectiveness of the mechanism on quality in low-and middle income countries. |
|  |  | Medicines registry | No reviews found | No reviews found | Insufficient review-level evidence to either support or discount the effectiveness of the mechanism on quality. |
|  |  | Price controls | No reviews found | No reviews found | Insufficient review-level evidence to either support or discount the effectiveness of the mechanism on quality. |
|  |  | Regulation of market entry (or market access) | Faden et al. found relatively little evidence on the effects of regulating market entry in low and middle income countries and as result was unable to make definitive conclusions.(73) | One older low-quality review | Insufficient review-level evidence to either support or discount the effectiveness of the mechanism on quality. |
|  |  | Medicine authentication system | Hamilton et al. found that registration of medicines with a competent authority is associated with higher medicine quality and reduced the risk of low-quality or counterfeit medicines from entering the system.(132)  Fadlallah et al. found no evidence to support the use of product authentication in reducing the prevalence of counterfeit medicines. However, included studies found that they may be effective in identifying counterfeit medicines once they are already in the supply chain. The authors noted that all studies including medicine authentication technologies remained in their pilot stage, so long-term outcomes remain unknown.(133) | One overview of systematic reviews and one medium-quality review. | Tentative review- level evidence to support the effectiveness of the mechanism on quality. |
|  |  | Standardized procurement processes | Hamilton et al. report on the importance of standardized procurement processes, providing examples of countries where these approaches have been implemented, but no findings of effectiveness were provided in the review.(132) | One recent overview of systematic reviews. | Insufficient review-level evidence to either support or discount the effectiveness of the mechanism on quality. |
|  | Process-oriented | Computerized order entry | Manias et al, found mixed results for computerized order entry as it was found in three of five studies to increase the total number of reported medication errors following implementation but reduced the number of errors that did harm to patients.(83)  Camire et al. found from three studies and that computerized order entry reduces the number of medication errors among elderly patients in the ICU.(90)  Lu et al. found in one systematic review that computerized order entry with clinical decision support improved prescribing and reduced medication error rates. (94)  Nuckols et al. found in a meta-analysis of 13 studies that computerized order entry was associated with a 50% reduction in preventable adverse drug events.(109) | One recent high-quality review and three older medium-quality reviews | Sufficient review-level evidence to support the effectiveness of the mechanism on quality |
|  |  | Pharmacy and treatment committees | No reviews found | No reviews found | Insufficient review-level evidence to either support or discount the effectiveness of the mechanism on quality. |
|  |  | Supply chain management | Hinrichs et al. found that collective approaches to procurement may lead to efficiencies and cost savings, however there was not sufficient evidence to make definitive conclusions.(134) |  | Insufficient review-level evidence to either support or discount the effectiveness of the mechanism on quality. |
|  |  | Audit of procurement processes | No reviews found | No reviews found | Insufficient review-level evidence to either support or discount the effectiveness of the mechanism on quality. |
|  |  | Medicines appearance checklist | Hamilton et al. described the use of medicines appearance checklists, but provided no evidence on the effectiveness of its use.(132) | One recent overview of systematic reviews. | Insufficient review-level evidence to either support or discount the effectiveness of the mechanism on quality. |
|  | Output-oriented | Pharmacovigilance (system, centres or committees) | Hamilton et al described pharmacovigilance reporting at the international level as being critical to ensuring the quality of medicines, however the effectiveness of this reporting was not examined.(132)  Fadlallah et al. found that pharmacovigilance systems have been able to correctly detect counterfeit and substandard medicine when implemented at the national level. However, authors not that under-reporting remains a critical mediator to the success of these systems.(133) | One recent overview of systematic reviews and one recent medium-quality systematic review. | Tentative review-level evidence to support the effectiveness of the mechanism on quality. |
|  | Outcome-oriented | Health Technology Assessment | Gagnon et al. found some positive results from the use of local hospital-based health technology assessments, both on hospital-decisions and budgets. However, evidence on three other types of local HTAs was inconclusive or less positive, for example mini-HTAs were found to rarely be used to inform decisions. Authors noted that relatively little evidence on the effectiveness of HTAs was available and more research in the area in required.(135) | One recent medium-quality review. | Insufficient review-level evidence to either support or discount the effectiveness of the mechanism on quality. |
| Esnuring adequate health facilities | Input-oriented | Changing facilities physical structure | Conry et al. found that technical interventions, which included changing facilities physical structures, were more effective than interpersonal interventions. However, the review did not identify specific effectiveness findings for changing physical functions. | One older medium-quality review. | Insufficient review-level evidence to either support or discount the effectiveness of the mechanism on quality. |
|  |  | Facility accreditation | Greenfield et al. found in four studies that facility accreditation promoted organizational change. However, mixed results were found on effects on quality of care from 18 studies. The review acknowledged that comparing the effectiveness of accreditation across facilities and across quality indicators may complicate the evidence base. Further, the review found no association in three studies between facility accreditation and patient satisfaction.(116)  Scott found no significant correlation between external facility accreditation, facility quality, and safety indicators.(69)  Brubakk et al. found no conclusions could be determined about the effectiveness of accreditation on hospital quality of care. Authors of the review pointed to the complex nature of the intervention and varied implementation across jurisdictions and hospitals.(136)  Petit Dit and Regnaux found mixed results and determined that no conclusions could be made about the effectiveness of magnet-accreditation status in the US on nursing or patient outcomes due to methodological limitations in the literature.(137)  Sutherland et al. found mixed evidence with regards to accreditation of healthcare facilities, with two studies finding improvements in quality while two additional studies found discrepancies between accreditation scores and measures of quality and safety. One additional study included in the same review found that accreditation improved compliance but had no significant improvement on overall quality. The same review included three studies that found inspections acted as a catalyst for improvement among regulated organizations.(82)  Hastings et al. found mixed evidence on the use of Magnet accreditation status among U.S. hospitals and found no conclusive evidence about their impact on quality of care but did find higher levels of job satisfaction among nurses working at accredited hospitals.(138)  Sharma et al. found that in a multi-country analysis of quality improvement efforts in labour and birth, accreditation was critical to ensuring high quality obstetrics care in low and middle income countries.(123) | Two older and one recent overview of systematic reviews, one recent high-quality review, one recent medium-quality, and two older low-quality reviews | Insufficient review-level evidence to either support or discount the effectiveness of the mechanism on quality, however was found in one review to be effective in low income countries. . |
|  |  | Facility certification | No reviews found. | No reviews found. | Insufficient review-level evidence to either support or discount the effectiveness of the mechanism on quality. |
|  |  | Facility standards | Berglas et al. did not directly examine the effects of facility standards on quality of care. | One recent medium-quality review | Insufficient review-level evidence to either support or discount the effectiveness of the mechanism on quality. |
|  |  | Facility-based safety protocols | No reviews found. | No reviews found. | Insufficient review-level evidence to either support or discount the effectiveness of the mechanism on quality. |
|  |  | Permits/permitting | No reviews found. | No reviews found. | Insufficient review-level evidence to either support or discount the effectiveness of the mechanism on quality. |
|  | Process-oriented | Facility inspections | Flodgren et al. were unable to determine the impact of facility inspections on health organizations behaviours, health professionals behaviours or patient outcomes from two studies included in a systematic review.(139) | One older high-quality review. | Insufficient review-level evidence to either support or discount the effectiveness of the mechanism on quality. |
|  | Output-oriented | Public reporting on performance by facilities | Scott found that public reporting exerted little effect on clinician’s or facility’s performance and were not readily accessed by consumers.(69)  Fung et al. found that public reporting by hospitals had no effect on patients’ choice of hospital but was associated with an increase in quality improvement activity at hospitals. The evidence on public reporting on effectiveness, safety and patient centeredness remains limited.(126)  Totten et al. found that organizations tended to make improvements to the quality of care based on public reports, however public reports were not used by patients to inform their choice of care organization. The review found that public reporting is more effective in competitive healthcare markets.(127)  Parker et al. found mixed results from the public reporting of quality metrics for strokes. Of 14 studies included, nine found positive associations between public reporting and patient outcomes, while the other five found no or very limited association.(129)  Behrendt et al. found some evidence that public reporting reduced access to surgery for high risk patients and those from different ethnic backgrounds, no evidence to support the use of public reporting as intrinsic motivation to improve their practice, and mixed findings regarding whether public reporting data is used to make referrals or decisions on commissioning.(130) | One older high-quality, one recent medium-quality and three older medium-quality reviews. | Tentative review-level evidence to support the effectiveness of the mechanism on quality, however one systematic review warns against the negative implact of public reporting on high-risk patients. . |
| Optimizing information systems | Input-oriented | Data protection and confidentiality protocols | No reviews found. | No reviews found. | Insufficient review-level evidence to either support or discount the effectiveness of the mechanism on quality. |
|  |  | Electronic health records | Campanella et al. found in 47 studies that when successfully implemented EHRs were associated with increases in efficiency, improved guideline adherence and reduced adverse events, however no association was found for mortality.(23)  McMurchy et al found in one systematic review that the use of electronic medical records has improved overall patient satisfaction with their visits, improve physician familiarity with patients and improve the comprehensiveness of decisions made during a patients’ visit.(46) | One recent medium-quality review and one older low-quality review. | Sufficient review-level evidence to support the effectiveness of the mechanism on quality. |
|  |  | Electronic patient registry | Cabello et al. found electronic patient registries were effective on improving diabetes self-management and processes of care in rural communities *(68)*.  Shojania et al. found some evidence that electronic patient registries improved diabetes outcomes, however also noted that despite accounting for other variables the implementation of electronic patient registries may indicate greater investment in quality improvement *(67)*. | One recent high-quality and one older medium-quality. | Tentative review-level evidence to support the effectiveness of the mechanism. |
|  |  | Unique patient identifier | No reviews found. | No reviews found. | Insufficient review-level evidence to either support or discount the effectiveness of the mechanism on quality. |

**References**

- 1. World Forum for Education. Accreditation 2018 [Available from: <http://wfme.org/accreditation/>.
- 2. Flottorp S, Jamtvedt G, Gibis B, McKee M. Using audit and feedback to health professionals to improve the quality and safety of health care. London: European Observatory on Health Systems and Policies; 2010.
- 3. Perri-Moore S, Kapsandoy S, Doyon K, Archer M, Shane-McWhorter L, Bray BE, et al. Automated Alerts and Reminders Targeting Patients: A Review of the Literature. Patient Educ Couns. 2016;99(6):953-9.
- 4. Pharmaceutical Bar Coding: Moving Forward in Canada. The Canadian Journal of Hospital Pharmacy. 2009;62(4):328-30.
- 5. World Health Organization, World Bank Group, Organization for Economic Cooperation and Development. Delivering quality health services: A global imperative for universal health coverage. Geneva; 2018.
- 6. Management Sciences for Health. Management Sciences for Health. Arlington; 2012.
- 7. Schrijvers G, van Hoorn A, Huiskes N. The care pathway: concepts and theories: an introduction. International journal of integrated care. 2012;12(Spec Ed Integrated Care Pathways):e192.
- 8. Conry MC, Humphries N, Morgan K, McGowan Y, Montgomery A, Vedhara K, et al. A 10 year (2000-2010) systematic review of interventions to improve quality of care in hospitals. BMC health services research. 2012;12:275.
- 9. World Health Organization. Laboratory quality management system. Geneva; 2011.
- 10. Street J, Duszynski K, Krawczyk S, Braunack-Mayer A. The use of citizens' juries in health policy decision-making: a systematic review. Social science & medicine (1982). 2014;109:1-9.
- 11. World Health Organization. WHO surgical safety checklist and implementation manual. Geneva; 2008.
- 12. U.S. National Library of Medicine. Collection Development Manual: Standards and Guidelines [Available from: <https://www.nlm.nih.gov/tsd/acquisitions/cdm/formats46.html>.
- 13. WHO health systems strengthening glossary. Geneva: World Health Organization; 2011.
- 14. Glossary of terms. The European Framework for Action on Integrated Health Services Delivery. Copenhagen: World Health Organization Regional Office for Europe; 2016.
- 15. Clinical Practice Guidelines: Directions for a New Program. In: Field MJ, Lohr KN, editors. Clinical Practice Guidelines: Directions for a New Program. Washington (DC)1990.
- 16. A glossary of terms for community health care and services for older persons. Geneva: World Health Organization; 2004.
- 17. Snowdon DA, Leggat SG, Taylor NF. Does clinical supervision of healthcare professionals improve effectiveness of care and patient experience? A systematic review. BMC health services research. 2017;17(1):786.
- 18. World Health Organization Western Pacific Region. Strengthening health workforce regulation in the Western Pacific Region. Manila; 2016.
- 19. World Health Organization, Institutional Repository for Information Sharing. Regional guidelines for continuing medical education (CME/CPD) activities India: World Health Organization,; 2010 [
- 20. Goncalves-Bradley DC, Lannin NA, Clemson LM, Cameron ID, Shepperd S. Discharge planning from hospital. The Cochrane database of systematic reviews. 2016(1):Cd000313.
- 21. Workman TA. Engaging Patients in Information Sharing and Data Collection. Rockville (MD) Agency for Healthcare Research and Quality; 2013.
- 22. O'Brien MA, Rogers S, Jamtvedt G, Oxman AD, Odgaard-Jensen J, Kristoffersen DT, et al. Educational outreach visits: effects on professional practice and health care outcomes. The Cochrane database of systematic reviews. 2007(4):Cd000409.
- 23. Campanella P, Lovato E, Marone C, Fallacara L, Mancuso A, Ricciardi W, et al. The impact of electronic health records on healthcare quality: a systematic review and meta-analysis. European journal of public health. 2016;26(1):60-4.
- 24. Workman TA. AHRQ Methods for Effective Health Care. Engaging Patients in Information Sharing and Data Collection: The Role of Patient-Powered Registries and Research Networks. Rockville (MD): Agency for Healthcare Research and Quality (US); 2013.
- 25. World Health Organization. Essential medicines and health products Geneva: World Health Organization; 2019 [Available from: <https://www.who.int/medicines/services/essmedicines_def/en/>.
- 26. McDonald KM, Chang C, Schultz E. AHRQ Methods for Effective Health Care. Through the Quality Kaleidoscope: Reflections on the Science and Practice of Improving Health Care Quality: Closing the Quality Gap: Revisiting the State of the Science. Rockville (MD): Agency for Healthcare Research and Quality (US); 2013.
- 27. Rooney AL, van Ostenberg PR. Licensure, accreditation, and certification: approaches to health services quality. Quality assurance methodology refinement series. Bethesda, MD: USAID; 1999.
- 28. Modern health care delivery systems, care coordination and the role of hospitals. Compiled report of the workshop organized by the Beligum Federal Public Health Service and WHO Europe, held in Brussels, Belgium, 21-22 November 2011, and the internal WHO expert meeting on roadmap developmetn, held in Copenhagen, Denmark, 12 January 2012. Copenhagen: World Health Organization Regional Office for Europe; 2012.
- 29. World health Organization Regional Office for Europe. How can hospital performance be measured and monitored? Geneva; 2003.
- 30. Institute of Medicine Committee on Quality of Health Care in America. To err is human: Building a safer health system. Kohn L, Corrigan J, Donaldson M, editors. Washington D.C.,: Institutes of Medicine,; 2000.
- 31. Agency for Healthcare Research And Quality. Glossary 2018 [Available from: <https://psnet.ahrq.gov/glossary/a>.
- 32. World Health Organization. Human resources for health information systems: Minimum data set for workforce registry. Geneva; 2015.
- 33. Ettorchi-Tardy A, Levif M, Michel P. Benchmarking: a method for continuous quality improvement in health. Healthcare policy = Politiques de sante. 2012;7(4):e101-19.
- 34. United Nations Economic Commission for Europe. Globally Harmonized System of Classification and Labelling of Chemicals Geneva: UNECE; 2018 [Available from: <http://www.unece.org/trans/danger/publi/ghs/ghs_welcome_e.html>.
- 35. Borgermans L, Langins M. Strengthening a competent health workforce for the provision of coordinated/integrated health services. Copenhagen: World Health Organization Regional Office for Europe; 2015.
- 36. Federal Drug Administration. FDA pre-approval inspection (PAI) program and how to prepare for a successful outcome. Washington, D.C.; 2015.
- 37. Naughton B, Roberts L, Dopson S, Chapman S, Brindley D. Effectiveness of medicines authentication technology to detect counterfeit, recalled and expired medicines: a two-stage quantitative secondary care study. BMJ open. 2016;6(12):e013837.
- 38. Federation IP. Tool for Visual Inspection of Medicines n.d.
- 39. Kobewka DM, van Walraven C, Turnbull J, Worthington J, Calder L, Forster A. Quality gaps identified through mortality review. BMJ Quality &amp; Safety. 2017;26(2):141-9.
- 40. Khalifa K, Ansari A, Violato C, Donnon T. Multisource feedback to assess surgical practice: A systematic review. Journal of Surgical Education 2013;70(4):475-86.
- 41. Zayyan M. Objective Structured Clinical Examination: The Assessment of Choice. Oman Medical Journal. 2011;26(4):219-22.
- 42. Hospital TO. Patient Decision Aids 2015 [Available from: <https://decisionaid.ohri.ca/>.
- 43. Lee R, Baeza JI, Fulop NJ. The use of patient feedback by hospital boards of directors: a qualitative study of two NHS hospitals in England. BMJ Quality &amp; Safety. 2018;27(2):103-9.
- 44. Organization for Economic Cooperation and Development. Caring for quality in health: Lessons learned from 15 reviews in health care quality. Paris; 2017.
- 45. Breen KJ. Revalidation - what is the problem and what are the possible solutions? The Medical journal of Australia. 2014;200(3):153-6.
- 46. McMurchy D. What are the critical attributes and benefits of a high-quality primary healthcare system? Ottawa; 2009.
- 47. Resolution: Strengthening people-centred health systems in the WHO European Region: framework for action on integrated health services delivery (2016).
- 48. Agency for Healthcare Research and Quality. Practice Facilitation Handbook: Creating quality improvement teams and QI plans 2013 [Available from: <https://www.ahrq.gov/professionals/prevention-chronic-care/improve/system/pfhandbook/mod14.html>.
- 49. Robert Wood-Johnson Foundation. Quality/Equality Glossary 2013 [Available from: <https://www.rwjf.org/en/library/research/2013/04/quality-equality-glossary.html>.
- 50. Agency for Healthcare Research and Quality. Patient Safety Network Washington D.C.: Agency for Healthcare Research and Quality; 2018 [Available from: [www.psnet.ahrq.gov](file:///Users/ericabarbazza/Downloads/www.psnet.ahrq.gov)
- 51. Gagliardi AR, Brouwers MC, Finelli A, Campbell CM, Marlow BA, Silver IL. Physician self-audit: a scoping review. The Journal of continuing education in the health professions. 2011;31(4):258-64.
- 52. Ferrer L. Engaging patients, carers and communities for the provision of coordinated/integrated health services: strategies and tools. Copenhagen: World Health Organization Regional Office for Europe; 2015.
- 53. World Health Organization. WHO simulation exercise manual. Geneva; 2017.
- 54. National Institute for Clinical and Evaluative Sciences. Medicines optimisation. Quality statement 5: Medicines reconciliation in primary care; 2016.
- 55. World Health Organization. Assuring medication accuracy at transitions in care: Medication reconciliation. Geneva; 2011.
- 56. USAID. The logistics handbook: A practical guide for the supply chain management of health commodities. Arlington; 2011.
- 57. World Health Organization. Task shifting: Rational redistribution o tasks among health workforce teams - global recommendations and guidelines. Geneva; 2008.
- 58. Burns PB, Rohrich RJ, Chung KC. The levels of evidence and their role in evidence-based medicine. Plastic and reconstructive surgery. 2011;128(1):305-10.
- 59. Sarrami-Foroushani P, Travaglia J, Debono D, Braithwaite J. Implementing strategies in consumer and community engagement in health care: results of a large-scale, scoping meta-review. BMC health services research. 2014;14:402.
- 60. Mitton C, Smith N, Peacock S, Evoy B, Abelson J. Public participation in health care priority setting: A scoping review. Health policy. 2009;91(3):219-28.
- 61. Kurtzman ET, Greene J. Effective presentation of health care performance information for consumer decision making: A systematic review. Patient Educ Couns. 2016;99(1):36-43.
- 62. Wagner C, van der Wal G, Groenewegen PP, de Bakker DH. The effectiveness of quality systems in nursing homes: a review. Quality in health care: QHC. 2001;10(4):211-7.
- 63. Nilsen ES, Myrhaug HT, Johansen M, Oliver S, Oxman AD. Methods of consumer involvement in developing healthcare policy and research, clinical practice guidelines and patient information material. The Cochrane database of systematic reviews. 2006(3):Cd004563.
- 64. Conklin A, Morris Z, Nolte E. What is the evidence base for public involvement in health-care policy?: results of a systematic scoping review. Health expectations : an international journal of public participation in health care and health policy. 2015;18(2):153-65.
- 65. Ranji S, Steinman M, Shojania K, Gonzales R. Interventions to reduce unnecessary antibiotic prescribing: A systematic review and quantitative analysis. Medical Care. 2008;46(8):847-62.
- 66. Walsh JM, McDonald KM, Shojania KG, Sundaram V, Nayak S, Lewis R, et al. Quality improvement strategies for hypertension management: a systematic review. Med Care. 2006;44(7):646-57.
- 67. Shojania KG, Ranji SR, McDonald KM, Grimshaw JM, Sundaram V, Rushakoff RJ, et al. Effects of quality improvement strategies for type 2 diabetes on glycemic control: a meta-regression analysis. Jama. 2006;296(4):427-40.
- 68. Ricci-Cabello I. Improving Diabetes Care in Rural Areas: A Systematic Review and Meta-Analysis of Quality Improvement Interventions in OECD Countries. 2013;8(12).
- 69. Scott I. What are the most effective strategies for improving quality and safety of health care? Internal medicine journal. 2009;39(6):389-400.
- 70. Rotter T, Kugler J, Kock R, Gothe H, Twork S, van Oostrum J, et al. A systematic review and meta-analysis of the effects of clinical pathways on length of stay, hospital costs and patient outcomes. BMC Health Service Research. 2008;8:265.
- 71. Ovretveit J. Do changes to patient-provider relationships improve quality and save money? London; 2012.
- 72. Colla CH, Mainor AJ, Hargreaves C, Sequist T, Morden N. Interventions Aimed at Reducing Use of Low-Value Health Services: A Systematic Review. Medical care research and review : MCRR. 2017;74(5):507-50.
- 73. Faden L, Vialle-Valentin C, Ross-Degnan D, Wagner A. Active pharmaceutical management strategies of health insurance systems to improve cost-effective use of medicines in low- and middle-income countries: a systematic review of current evidence. Health policy. 2011;100(2-3):134-43.
- 74. Patil S, Ruppar T, Koopman R, Lindbloom E, Elliot S, Mehr D. Peer support interventions for adults with diabetes: A meta-analysis of hemoglobin AIc outcomes. Annals of Family Medicine,. 2016;14(6):540-51.
- 75. Dale J, Caramlau I, Lindenmeyer A, Williams S. Peer support telephone calls for improving health. Cochrane Database of Systematic Reviews. 2008;4:1-44.
- 76. Ryan R, Santesso N, Lowe D, Hill S, Grimshaw J, Prictor M, et al. Interventions to improve safe and effective medicines use by consumers: an overview of systematic reviews. The Cochrane database of systematic reviews. 2014(4):Cd007768.
- 77. Cheraghi-Sohi S, Bower P. Can the feedback of patient assessments, brief training, or their combination, improve the interpersonal skills of primary care physicians? A systematic review. BMC health services research. 2008;8:179.
- 78. Ridd M, Shaw A, Lewis G, Salisbury C. The patient-doctor relationship: a synthesis of the qualitative literature on patients' perspectives. The British journal of general practice : the journal of the Royal College of General Practitioners. 2009;59(561):e116-33.
- 79. Kotronoulas G, Kearney N, Maguire R, Harrow A, Di Domenico D, Croy S, et al. What is the value of the routine use of patient-reported outcome measures toward improvement of patient outcomes, processes of care, and health service outcomes in cancer care? A systematic review of controlled trials. Journal of clinical oncology : official journal of the American Society of Clinical Oncology. 2014;32(14):1480-501.
- 80. Almeida RS, Bourliataux-Lajoinie S, Martins M. Satisfaction measurement instruments for healthcare service users: a systematic review. Cadernos de saude publica. 2015;31(1):11-25.
- 81. Hughes R, editor. Patient safety and quality: An evidence-based handbook for nurses Rockville, U.S.: Agency for Healthcare Research and Quality; 2008.
- 82. Sutherland K, Leatherman S. Regulation and quality improvement: A review of the evidence. London; 2006.
- 83. Manias E, Williams A, Liew D. Interventions to reduce medication errors in adult intensive care: a systematic review. British journal of clinical pharmacology. 2012;74(3):411-23.
- 84. Willey B, Paintain L, Mangham L, Car J, Amstarong Schellenberg J. Effectiveness of interventions to strengthen national health service delivery on coverage, access,quality and equity in the use of health services in low and lower middle income countries. London: EPPI-Centre 2012.
- 85. Ament SM, de Groot JJ, Maessen JM, Dirksen CD, van der Weijden T, Kleijnen J. Sustainability of professionals' adherence to clinical practice guidelines in medical care: a systematic review. BMJ open. 2015;5(12):e008073.
- 86. White DE, Straus SE, Stelfox HT, Holroyd-Leduc JM, Bell CM, Jackson K, et al. What is the value and impact of quality and safety teams? A scoping review. Implementation science : IS. 2011;6:97.
- 87. Dayal P, Hort K. Policy brief: Quality of care - What are effective policy options for governments in low- and middle-income countries to improve and regulate the quality of ambulatory care? . Manila, Phillipines: Asia Pacific Observatory on Health Systems and Policies; 2015.
- 88. Lugtenberg M, Burgers JS, Westert GP. Effects of evidence-based clinical practice guidelines on quality of care: a systematic review. Quality & safety in health care. 2009;18(5):385-92.
- 89. Lyons V, Popejoy L. Meta-analysis of surgical safety checklist effects on teamwork, communication, morbidity, mortality and safety. West Jounral of Nursing Research. 2013;36(2):245-61.
- 90. Camire E, Moyen E, Stelfox HT. Medication errors in critical care: risk factors, prevention and disclosure. CMAJ : Canadian Medical Association journal = journal de l'Association medicale canadienne. 2009;180(9):936-43.
- 91. Marcum ZA, Handler SM, Wright R, Hanlon JT. Interventions to improve suboptimal prescribing in nursing homes: A narrative review. The American journal of geriatric pharmacotherapy. 2010;8(3):183-200.
- 92. Sketris IS, Langille Ingram EM, Lummis HL. Strategic opportunities for effective optimal prescribing and medication management. The Canadian journal of clinical pharmacology = Journal canadien de pharmacologie clinique. 2009;16(1):e103-25.
- 93. Robertson J, Walkom E, Pearson SA, Hains I, Williamsone M, Newby D. The impact of pharmacy computerised clinical decision support on prescribing, clinical and patient outcomes: a systematic review of the literature. The International journal of pharmacy practice. 2010;18(2):69-87.
- 94. Lu CY, Ross-Degnan D, Soumerai SB, Pearson SA. Interventions designed to improve the quality and efficiency of medication use in managed care: a critical review of the literature - 2001-2007. BMC health services research. 2008;8:75.
- 95. McDonald K, Matesic B, Contopoulos-Ioannidis D, Lonhart J, Schmidt E, Pineda N. Patient safety strategies targeted at diagnostic error: A systematic review. Annals of Internal Medicine. 2013;158(5):381-9.
- 96. Laugaland K, Aese K, Barach P. Interventions to improve patient safety in transitional care - A review o the evidence. Work. 2012;41(Suppl 1):2915-24.
- 97. Christensen M, Lundh A. Medication review in hospitalised patients to reduce morbidity and mortality. The Cochrane database of systematic reviews. 2016;2:Cd008986.
- 98. Hammad EA, Bale A, Wright DJ, Bhattacharya D. Pharmacy led medicine reconciliation at hospital: A systematic review of effects and costs. Research in social & administrative pharmacy : RSAP. 2017;13(2):300-12.
- 99. Chaudhuri D, Montgomery A, Gulenchyn K, Joseph P. Effectiveness of quality improvement interventions at reducing inappropriate cardiac imaging: A systematic review and meta-analysis. Circulation: Cardiovascular Quality and Outcomes. 2016;9:7-13.
- 100. Obeirne M, Oelke ND, Sterling P, Lait J, Zwicker K, Lewanczuk R, et al. A synthesis of quality improvement and accreditation mechanisms in primary healthcare. Ottawa; 2012.
- 101. Sykes MJ, McAnuff J, Kolehmainen N. When is audit and feedback effective in dementia care? A systematic review. International journal of nursing studies. 2018;79:27-35.
- 102. Brunsveld-Reinders AH, Arbous MS, De Vos R, De Jonge E. Incident and error reporting systems in intensive care: a systematic review of the literature. International journal for quality in health care : journal of the International Society for Quality in Health Care. 2016;28(1):2-13.
- 103. Bal G, David S, Sellier E, Francois P. [Value of morbidity and mortality review conferences for physician education and improvement of care quality and safety: a literature review]. Presse medicale (Paris, France : 1983). 2010;39(2):161-8.
- 104. McQueen C, Smyth M, Fisher J, Perkins G. Does the use of dedicated dispatch criteria by Emergency Medical Services optimise appropriate allocation of advanced care resources in cases of high severity trauma? A systematic review. Injury. 2015;46(7):1197-206.
- 105. Bigham BL, Buick JE, Brooks SC, Morrison M, Shojania KG, Morrison LJ. Patient safety in emergency medical services: a systematic review of the literature. Prehospital emergency care : official journal of the National Association of EMS Physicians and the National Association of State EMS Directors. 2012;16(1):20-35.
- 106. Synnot A, Karlsson A, Brichko L, Chee M, Fitzgerald M, Misra MC, et al. Prehospital notification for major trauma patients requiring emergency hospital transport: A systematic review. Journal of evidence-based medicine. 2017;10(3):212-21.
- 107. Bost N, Crilly J, Wallis M, Patterson E, Chaboyer W. Clinical handover of patients arriving by ambulance to the emergency department - a literature review. International emergency nursing. 2010;18(4):210-20.
- 108. Snyder SR, Favoretto AM, Derzon JH, Christenson RH, Kahn SE, Shaw CS, et al. Effectiveness of barcoding for reducing patient specimen and laboratory testing identification errors: a Laboratory Medicine Best Practices systematic review and meta-analysis. Clinical biochemistry. 2012;45(13-14):988-98.
- 109. Nuckols TK, Smith-Spangler C, Morton SC, Asch SM, Patel VM, Anderson LJ, et al. The effectiveness of computerized order entry at reducing preventable adverse drug events and medication errors in hospital settings: a systematic review and meta-analysis. Systematic reviews. 2014;3:56.
- 110. Schouten L, Hulscher M, van Everdingen J, Huijsman R, Grol R. Evidence for the impact of quality improvement collaboratives: Systematic review. British Medical Journal. 2008;336:1491-4.
- 111. Wells S, Tamir O, Gray J, Naidoo D, Bekhit M, Goldmann D. Are quality improvement collaboratives effective? A systematic review. BMJ quality & safety. 2018;27(3):226-40.
- 112. Phillips CB, Pearce CM, Hall S, Travaglia J, de Lusignan S, Love T, et al. Can clinical governance deliver quality improvement in Australian general practice and primary care? A systematic review of the evidence. The Medical journal of Australia. 2010;193(10):602-7.
- 113. de Vos M, Graafmans W, Kooistra M, Meijboom B, Van Der Voort P, Westert G. Using quality indicators to improve hospital care: a review of the literature. International journal for quality in health care : journal of the International Society for Quality in Health Care. 2009;21(2):119-29.
- 114. Nicolay CR, Purkayastha S, Greenhalgh A, Benn J, Chaturvedi S, Phillips N, et al. Systematic review of the application of quality improvement methodologies from the manufacturing industry to surgical healthcare. The British journal of surgery. 2012;99(3):324-35.
- 115. Kurji Z, Premani ZS, Mithani Y. Review and analysis of quality healthcare system enhancement in developing countries. JPMA The Journal of the Pakistan Medical Association. 2015;65(7):776-81.
- 116. Greenfield D, Braithwaite J. Health sector accreditation research: a systematic review. International journal for quality in health care : journal of the International Society for Quality in Health Care. 2008;20(3):172-83.
- 117. Dieleman M, Gerretsen B, van der Wilt G. Human resource management interventions to improve health workers’ performance in low and middle income countries: A reality review. Health Research Policy and Systems. 2009;7(7).
- 118. Murdoch NL, Bottorff JL, McCullough D. Simulation education approaches to enhance collaborative healthcare: a best practices review. International journal of nursing education scholarship. 2014;10.
- 119. Dilaveri CA, Szostek JH, Wang AT, Cook DA. Simulation training for breast and pelvic physical examination: a systematic review and meta-analysis. BJOG : an international journal of obstetrics and gynaecology. 2013;120(10):1171-82.
- 120. Gordon M, Darbyshire D, Baker P. Non-technical skills training to enhance patient safety: a systematic review. Medical education. 2012;46(11):1042-54.
- 121. Craig S. Direct observation of clinical practice in emergency medicine education. Academic emergency medicine : official journal of the Society for Academic Emergency Medicine. 2011;18(1):60-7.
- 122. Dieleman M, Harnmeijer J. Improving health worker performance: In search of promising practices. Geneva; 2006.
- 123. Sharma G, Mathai M, Dickson KE, Weeks A, Hofmeyr G, Lavender T, et al. Quality care during labour and birth: a multi-country analysis of health system bottlenecks and potential solutions. BMC pregnancy and childbirth. 2015;15 Suppl 2:S2.
- 124. Cervero RM, Gaines JK. The impact of CME on physician performance and patient health outcomes: an updated synthesis of systematic reviews. The Journal of continuing education in the health professions. 2015;35(2):131-8.
- 125. Setyonugroho W, Kennedy KM, Kropmans TJ. Reliability and validity of OSCE checklists used to assess the communication skills of undergraduate medical students: A systematic review. Patient Educ Couns. 2015.
- 126. Fung CH, Lim YW, Mattke S, Damberg C, Shekelle PG. Systematic review: the evidence that publishing patient care performance data improves quality of care. Ann Intern Med. 2008;148(2):111-23.
- 127. Totten AM, Wagner J, Tiwari A, O'Haire C, Griffin J, Walker M. Closing the quality gap: revisiting the state of the science (vol. 5: public reporting as a quality improvement strategy). Evidence Report/Technology Assessment. 2012(2085):1-645.
- 128. Herrera CA, Lewin S, Paulsen E, Ciapponi A, Opiyo N, Pantoja T, et al. Governance arrangements for health systems in low-income countries: an overview of systematic reviews. The Cochrane database of systematic reviews. 2017;9(9):CD011085-CD.
- 129. Parker C, Schwamm LH, Fonarow GC, Smith EE, Reeves MJ. Stroke quality metrics: systematic reviews of the relationships to patient-centered outcomes and impact of public reporting. Stroke. 2012;43(1):155-62.
- 130. Behrendt K, Groene O. Mechanisms and effects of public reporting of surgeon outcomes: A systematic review of the literature. Health policy. 2016;120(10):1151-61.
- 131. Young J, Slebodnik M, Sands L. Bar code technology and medication administration error. Journal of patient safety. 2010;6(2):115-20.
- 132. Hamilton WL, Doyle C, Halliwell-Ewen M, Lambert G. Public health interventions to protect against falsified medicines: a systematic review of international, national and local policies. Health policy and planning. 2016;31(10):1448-66.
- 133. Fadlallah R, El-Jardali F, Annan F, Azzam H, Akl EA. Strategies and Systems-Level Interventions to Combat or Prevent Drug Counterfeiting: A Systematic Review of Evidence Beyond Effectiveness. Pharmaceutical medicine. 2016;30(5):263-76.
- 134. Hinrichs S, Jahagirdar D, Miani C, Guerin B, Nolte E. Health Services and Delivery Research. Learning for the NHS on procurement and supply chain management: a rapid evidence assessment. Southampton (UK): NIHR Journals Library
- 135. Gagnon MP, Desmartis M, Poder T, Witteman W. Effects and repercussions of local/hospital-based health technology assessment (HTA): a systematic review. Systematic reviews. 2014;3:129.
- 136. Brubakk K, Vist GE, Bukholm G, Barach P, Tjomsland O. A systematic review of hospital accreditation: the challenges of measuring complex intervention effects. BMC health services research. 2015;15:280.
- 137. Petit Dit Dariel O, Regnaux JP. Do Magnet(R)-accredited hospitals show improvements in nurse and patient outcomes compared to non-Magnet hospitals: a systematic review. JBI database of systematic reviews and implementation reports. 2015;13(6):168-219.
- 138. Hastings SE, Armitage GD, Mallinson S, Jackson K, Suter E. Exploring the relationship between governance mechanisms in healthcare and health workforce outcomes: a systematic review. BMC health services research. 2014;14:479.
- 139. Flodgren G, Pomey M-P, Taber SA, Eccles MP. Effectiveness of external inspection of compliance with standards in improving healthcare organisation behaviour, healthcare professional behaviour or patient outcomes. The Cochrane database of systematic reviews. 2011(11):CD008992-CD.
